# Inhibition of the immunoproteasome modulates innate immunity to ameliorate muscle pathology of dysferlin-deficient BlAJ mice

**DOI:** 10.1038/s41419-022-05416-1

**Published:** 2022-11-19

**Authors:** A. Farini, L. Tripodi, C. Villa, F. Napolitano, F. Strati, D. Molinaro, F. Facciotti, B. Cassani, Y. Torrente

**Affiliations:** 1 Neurology Unit, Fondazione IRCCS Ca’ Granda Ospedale Maggiore Policlinico, Milan, Italy; 2 Stem Cell Laboratory, Dino Ferrari Center, Department of Pathophysiology and Transplantation, University of Milan, Milan, Italy; 3 Laboratorio di Chimica Clinica e Microbiologia, Fondazione IRCCS Cà Granda Ospedale Maggiore Policlinico, Milano, Italy; 4 Department of Experimental Oncology, European Institute of Oncology IRCCS, Milan, Italy; 5 Department of Biotechnology and Biosciences, University of Milano-Bicocca, Milan, Italy; 6 Department of Medical Biotechnologies and Translational Medicine, Università Degli Studi di Milano, 20089 Milan, Italy; 7 Humanitas Clinical and Research Center IRCCS, Rozzano, 20089 Milan, Italy

**Keywords:** Peritoneal macrophages, Chronic inflammation

## Abstract

Muscle repair in dysferlinopathies is defective. Although macrophage (Mø)-rich infiltrates are prominent in damaged skeletal muscles of patients with dysferlinopathy, the contribution of the immune system to the disease pathology remains to be fully explored. Numbers of both pro-inflammatory M1 Mø and effector T cells are increased in muscle of dysferlin-deficient BlAJ mice. In addition, symptomatic BlAJ mice have increased muscle production of immunoproteasome. In vitro analyses using bone marrow-derived Mø of BlAJ mice show that immunoproteasome inhibition results in C3aR1 and C5aR1 downregulation and upregulation of M2-associated signaling. Administration of immunoproteasome inhibitor ONX-0914 to BlAJ mice rescues muscle function by reducing muscle infiltrates and fibro-adipogenesis. These findings reveal an important role of immunoproteasome in the progression of muscular dystrophy in BlAJ mouse and suggest that inhibition of immunoproteasome may produce therapeutic benefit in dysferlinopathy.

## Introduction

Mutations in dysferlin gene (DYSF, MIM*603009) are responsible for recessively inherited dysferlinopathy which is most pronounced in the pelvic and shoulder girdle muscles (Limb girdle muscular dystrophy R2-LGMDR2, formerly LGMD2B), or distal myopathy with onset in gastrocnemius and soleus muscles in cases of Miyoshi myopathy (MM or MMD1), or distal myopathy with onset in the tibialis anterior (DMAT) (also referred to as DACM for distal anterior compartment myopathy) [1, 2]. Dysferlin is a transmembrane proteins, that is implicated in protein vesicle fusion and trafficking [3]: it is prevalently expressed in skeletal muscle but it is also present in macrophages (Mø), adipocytes, smooth muscle cells [4]. Dysferlin also interacts with Ca^2+^ handling proteins for excitation-contraction (EC) coupling at the transverse-tubules (T-tubules) in skeletal muscle [5, 6]. Moreover, dysferlin was detected in blood vessels and dysferlin-null mice displayed impaired angiogenic response compared to control mice [7]. LGMDR2 muscles are characterized by enhanced infiltration of macrophages and CD4+ T-cells in the perimysium [8] and the involvement of innate immune system [9–11].

The complement immune system including its activated anaphylatoxins, C3a and C5a, facilitate innate immune response [12]. Both C3a and C5a mediate vasodilation, increased vascular permeability, chemotaxis, and inflammation by innate immune cells through interaction with their specific receptors (C3aR, C5aR) [13]. Murine C3aR was mainly detected on Mø, but not on circulating neutrophils, T cells, and B cells [14], highlighting the potential of anti-inflammatory properties of C3a/C3aR axis. Consistently, C3a receptor signaling has been reported to be involved in Mø recruitment and muscle regeneration [15]. In addition, C3aR expression in aortic tissues confers protection from atherosclerosis through modulation of Mø toward the anti-inflammatory phenotype [16]. Muscle fibers of both animal models and LGMDR2 patients present abnormal activation of complement factors C4 and C5 together with the downregulation of the complement inhibitory factor CD55, the upregulation of major histocompatibility complex I (MHC-I) and the formation of the membrane attack complex (MAC, C5b-9) on their surface [11, 17, 18]. The lack of CD55 enhances the susceptibility of skeletal muscle to complement attack [19], leading to over-expression of inflammatory pathways dependent on heat shock proteins and HMGB1 [20]. This scenario is worsened by HMGB1 secretion from necrotic cells and by activation of macrophages toward a pro-inflammatory phenotype through a HMGB1-C1q signaling [21, 22]. Indeed, C1q can bind to PTX3 to activate the classical component cascade and together modulate Mø M1/M2 polarization [23]. Moreover, complement can enhance the release of metalloproteinases (MMPs) [24] and favor the expression of MMP2 through the C3a-C3aR complex [25].

To identify additional elements that influence the inflammatory response in dysferlinopathy, we focused on the immunoproteasome (IP), which is specifically involved in inflammatory responses, including cytokine production and antigen processing for presentation on MHC-I [26, 27]. Proteasomes of eukaryotic cells have a 20S constitutive core (c-20S) that contains the catalytic subunits β5, β1 and β2, accounting for chymotrypsin-, caspase- and trypsin-like activities, respectively [28]. However, haematopoietic cells as lymphocytes and monocytes as well as cells exposed to cytokines such as IFN-γ [29], express variable proportions of IP (i-20S) in which catalytic subunits are encoded by homologous genes that code for PSMB8 (formerly LMP7), PSMB9 (formerly LMP2) and multicatalytic endopeptidase complex subunit-1 (MECL-1) proteins [30]. IP subunits participate in generating MHC-I ligands, establishing the naive CD8+ T cell repertoire and shaping cytotoxic T cell response [31–33].

FDA-approved proteasome inhibitors (bortezomib, carfilzomib and ixazomib) comparably target both c-20S and i-20S to inhibits cytokine production in vitro and inflammation in vivo [34, 35]; however, most of proteasome inhibitors have considerable side effects that probably limit their clinical utility in chronic inflammatory diseases. ONX-0914 is a potent inhibitor specific for the highly active PSMB8 [36]: preclinical studies utilizing this compound demonstrated the therapeutic potential of IP inhibition in several inflammatory disorders [36, 37]. Furthermore, the ONX-0914-analog KZR has recently entered clinical trials for treatment of patients with autoimmune-triggered inflammation [36–39].

We previously demonstrated the therapeutical potential of IP inhibition in a murine animal model of Duchenne Muscular Dystrophy (DMD) [40, 41]. Here we aimed to examine the functional significance of ONX-0914 in BlAJ animal model of dysferlinopathy. In vitro treatment of peritoneal Mø from BlAJ mice with ONX-0914 resulted in C3aR1 and C5aR1 downregulation and upregulation of M2-associated signaling. Remarkably, IP inhibition ameliorates muscular dystrophy in BlAJ mice reducing C3 serum levels and promoting M2 Mø polarization. In addition, ONX-0914-treated muscles have significantly increased number of vessels, most of them expressing C3aR1 and C5aR1. All these data confirmed that both complement and macrophages interact closely to maintain the process of angiogenesis [42, 43] and suggest that IP inhibition trigger a cascade of events leading to M2 Mø polarization, reduction of muscle inflammation and macrophage-mediated vessel stability with consequent amelioration of muscle performance in dysferlinopathic BlAJ mice.

## Results

### The active phase of dysferlinopathy correlates with sustained innate immunity

**Fig. 1:**
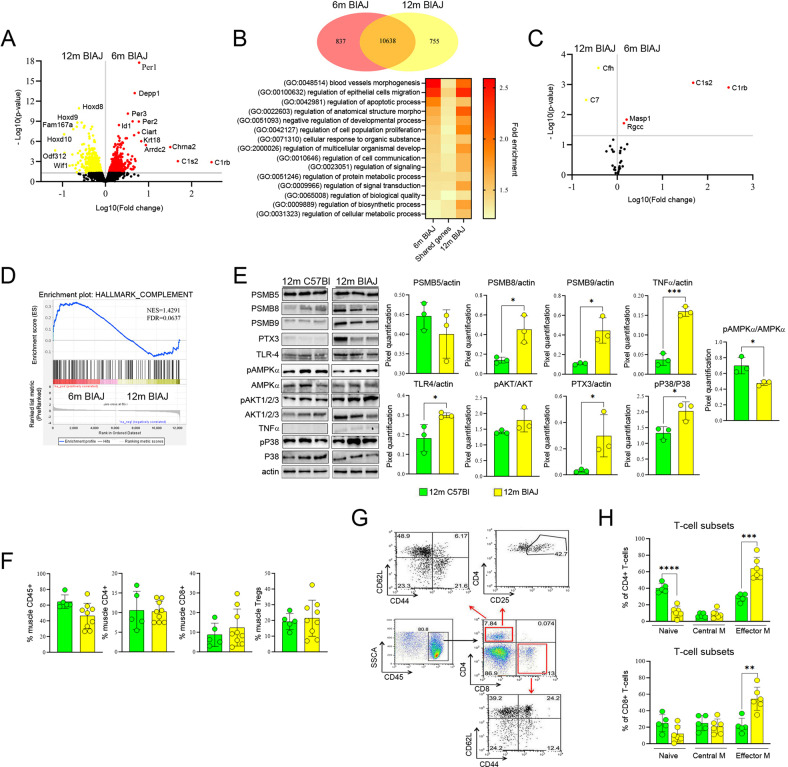
Inflammatory features of murine dysferlinopathic skeletal muscles. **A** Volcano plot analysis of genes differentially expressed in 6 m BlAJ vs 12 m BlAJ muscles: red and yellow dots represent genes upregulated in 6 m BlAJ and 12 m BlAJ, respectively, with a *p* value < 0.05 (corresponding to a -Log_10_(*p* value) > 1.30). **B** Gene ontology (GO) pathways enrichment in 6 m BlAJ and 12 m BlAJ mice, or both. For GO analysis 837 genes upregulated in 6 m BlAJ, 755 gene upregulated in 12 m BlAJ and 10,638 non-differentially expressed genes (based on RNA-seq data) were used. Only significant pathways (*p* value < 0.05) are shown. Redundant pathways are omitted from the figure. **C** Volcano plot analysis of genes involved in complement activation (Biological process; GO: 0006956) and complement receptor activity (Molecular function; GO: 000487). Red and yellow dots represent genes upregulated in 6 m BlAJ and 12 m BlAJ, respectively, with a *p* value < 0.05 (corresponding to a -Log_10_(*p* value) > 1.30). **D** GSEA plot for the annotated dataset “Hallmark_Complement” by the Molecular Signatures Database (MSigDB). Geneset size: 143; Normalized enrichment score (NES): 1.43; False discovery rate (FDR) *q* value: 0.064. **E** WB expression of IP subunits and inflammatory mediators in psoas muscle of 12 mo BlAJ and 12 mo C57Bl mice. **F** Evaluation of CD45+ cells, CD4+ and CD8+ T-lymphocytes and Tregs in skeletal muscles of 12 m BlAJ and C57Bl mice. **G** Representative FACS of CD4 and CD8 cell subpopulation profiles. The numbers within the panels indicate the percentage of each population of live cells, a gate of CD45-positive events defined infiltrating inflammatory cells. Within the CD45+ cells, two populations were separated by the expression levels of CD4 or CD8, whose subpopulations were separated according to CD44 and CD62L.Within CD4+ cell population, positivity for CD25 was also determined. **H** Evaluation of T-cell subsets—naive, central memory (CM), effector memory (EM)—expressing CD4 and/or CD8 in the muscles of 12 m C57Bl and 12 m BlAJ. Data are presented as mean ± SD of *n* = 3 independent experiments with *n* = 3–12 animals/group. One-way ANOVA and Student *t*-test: *p < 0.05, **p < 0.01, ***p < 0.001 and ****p < 0.0001.

Previous observations of psoas muscle isolated from the BlAJ mice younger than 6 months (6 m) of age showed little or no muscle pathology whereas inflammatory infiltration was observed in muscle isolated from 12-month olds (12 m) [44–46]. To understand the molecular mechanisms of the age-dependent muscle inflammation in dysferlin-deficient BlAJ mouse, we analyzed transcriptomic data generated from psoas muscles of pre-symptomatic 6 m and symptomatic 12 m BlAJ mice (Fig. 1A–C). The 6 m and 12 m muscles were molecularly distinct (Fig. 1A and Supplementary Table 1), with 837 genes upregulated in 6 m (including *Per1, Per2, Per3, Depp, Chrna2, C1s2* and *C1rb*) and 755 genes upregulated in 12 m (including *C7*, *Cfh* and *Homeobox* genes) (Tables 1 and 2).

**Table 1:** Genes upregulated in **A** 6 m and **B** 12 m BlAJ mice.

ENSEMBL ID	Symbol	Name	log2FoldChange	*p* value
*(A) Top 20 upregulated genes in 6* *m BlAJ mouse*				
ENSMUSG00000098470	C1rb	Complement Component 1, R Subcomponent B	8.182	1.25E–03
ENSMUSG00000079343	C1s2	Complement Component 1, S Subcomponent 2	5.587	8.72E–04
ENSMUSG00000022041	Chrna2	Cholinergic Receptor, Nicotinic, Alpha Polypeptide 2 (Neuronal)	4.996	7.10E–06
ENSMUSG00000002910	Arrdc2	Arrestin Domain Containing 2	3.116	3.48E–06
ENSMUSG00000023043	Krt18	Keratin 18	2.756	9.37E–07
ENSMUSG00000020893	Per1	Period Circadian Clock 1	2.591	1.75E–18
ENSMUSG00000055866	Per2	Period Circadian Clock 2	2.572	1.08E–09
ENSMUSG00000038550	Ciart	Circadian Associated Repressor Of Transcription	2.556	5.08E–08
ENSMUSG00000026205	Slc23a3	Solute Carrier Family 23 (Nucleobase Transporters), Member 3	2.318	8.21E–04
ENSMUSG00000064147	Rab44	Rab44, Member Ras Oncogene Family	2.266	4.35E–04
ENSMUSG00000048489	Depp1	Depp1 Autophagy Regulator	2.253	6.11E–14
ENSMUSG00000097391	Mirg	Mirna Containing Gene	2.247	7.28E–03
ENSMUSG00000037411	Serpine1	Serine (Or Cysteine) Peptidase Inhibitor, Clade E, Member 1	2.206	1.19E–07
ENSMUSG00000030329	Pianp	Pilr Alpha Associated Neural Protein	2.150	5.47E–03
ENSMUSG00000035042	Ccl5	Chemokine (C-C Motif) Ligand 5	2.146	1.35E–02
ENSMUSG00000059824	Dbp	D Site Albumin Promoter Binding Protein	2.088	1.03E–09
ENSMUSG00000026819	Slc25a25	Solute Carrier Family 25 (Mitochondrial Carrier, Phosphate Carrier), Member 25	2.059	1.65E–04
ENSMUSG00000024526	Cidea	Cell Death-Inducing Dna Fragmentation Factor, Alpha Subunit-Like Effector A	2.035	1.50E–02
ENSMUSG00000109372	Gm19410	Predicted Gene, 19410	1.973	5.03E–04
ENSMUSG00000021268	Meg3	Maternally Expressed 3	1.949	1.27E–02
*(B) Top 20 upregulated genes in 12* *m BlAJ mouse*				
ENSMUSG00000035963	Odf3l2	Outer Dense Fiber Of Sperm Tails 3-Like 2	–3.882	2.21E–05
ENSMUSG00000050368	Hoxd10	Homeobox D10	–3.193	8.88E–08
ENSMUSG00000021815	Mss51	Mss51 Mitochondrial Translational Activator	–3.169	5.30E–05
ENSMUSG00000020218	Wif1	Wnt Inhibitory Factor 1	–2.774	4.24E–03
ENSMUSG00000031737	Irx5	Iroquois Homeobox 5	–2.557	9.00E–05
ENSMUSG00000019932	Kera	Keratocan	–2.548	3.55E–03
ENSMUSG00000035095	Fam167a	Family With Sequence Similarity 167, Member A	–2.493	1.52E–08
ENSMUSG00000047443	Erfe	Erythroferrone	–2.321	1.06E–02
ENSMUSG00000079105	C7	Complement Component 7	–2.282	3.26E–03
ENSMUSG00000063953	Amd2	S-Adenosylmethionine Decarboxylase 2	–2.221	4.00E–02
ENSMUSG00000050069	Grem2	Gremlin 2, Dan Family Bmp Antagonist	–2.218	6.39E–09
ENSMUSG00000043342	Hoxd9	Homeobox D9	–2.174	1.53E–09
ENSMUSG00000034584	Exph5	Exophilin 5	–2.130	1.36E–03
ENSMUSG00000031250	Tnmd	Tenomodulin	–2.126	5.56E–05
ENSMUSG00000031673	Cdh11	Cadherin 11	–2.117	1.78E–03
ENSMUSG00000027102	Hoxd8	Homeobox D8	–2.049	1.15E–11
ENSMUSG00000026051	Ecrg4	Ecrg4 Augurin Precursor	–2.000	2.03E–02
ENSMUSG00000059493	Nhs	Nhs Actin Remodeling Regulator	–1.978	2.62E–05
ENSMUSG00000031906	Smpd3	Sphingomyelin Phosphodiesterase 3, Neutral	–1.929	7.39E–04
ENSMUSG00000057722	Lepr	Leptin Receptor	–1.867	1.15E–04

**Table 2:** List of genes involved in complement activation and complement receptor activity.

ENSEMBL ID	Symbol	Name	log2FoldChange	*p* value
*Upregulated genes in 6* *m BlAJ mouse*				
ENSMUSG00000098470	C1rb	Complement Component 1, R Subcomponent B	8.182263074	0.001250885
ENSMUSG00000079343	C1s2	Complement Component 1, S Subcomponent 2	5.587182004	0.000871793
ENSMUSG00000023992	Trem2	Triggering Receptor Expressed On Myeloid Cells 2	0.733157851	0.096146313
ENSMUSG00000022887	Masp1	Mannan-Binding Lectin Serine Peptidase 1	0.69300433	0.014662732
ENSMUSG00000073418	C4b	Complement Component 4B (Chido Blood Group)	0.533914616	0.140061036
ENSMUSG00000022018	Rgcc	Regulator Of Cell Cycle	0.49190354	0.019297844
ENSMUSG00000074361	C5ar2	Complement Component 5A Receptor 2	0.401272021	0.519986508
ENSMUSG00000024164	C3	Complement Component 3	0.295320134	0.346800669
ENSMUSG00000032679	Cd59a	Cd59A Antigen	0.285061323	0.125542455
ENSMUSG00000018446	C1qbp	Complement Component 1, Q Subcomponent Binding Protein	0.227746982	0.16028519
ENSMUSG00000036905	C1qb	Complement Component 1, Q Subcomponent, Beta Polypeptide	0.220271038	0.391784631
ENSMUSG00000038845	Phb	Prohibitin	0.213011255	0.187137225
ENSMUSG00000049130	C5ar1	Complement Component 5A Receptor 1	0.201042989	0.598542063
ENSMUSG00000036887	C1qa	Complement Component 1, Q Subcomponent, Alpha Polypeptide	0.16729138	0.520117499
ENSMUSG00000042436	Mfap4	Microfibrillar-Associated Protein 4	0.145458769	0.659127548
ENSMUSG00000036896	C1qc	Complement Component 1, Q Subcomponent, C Chain	0.140524774	0.583171884
ENSMUSG00000001128	Cfp	Complement Factor Properdin	0.090370496	0.776127139
ENSMUSG00000024371	C2	Complement Component 2 (Within H-2S)	0.062776152	0.881526526
ENSMUSG00000015083	C8g	Complement Component 8, Gamma Polypeptide	0.06103822	0.847147746
*Upregulated genes in 12* *m BlAJ mouse*				
ENSMUSG00000038521	C1s1	Complement Component 1, S Subcomponent 1	–0.115238369	0.86477287
ENSMUSG00000040552	C3ar1	Complement Component 3A Receptor 1	–0.153607904	0.684275666
ENSMUSG00000055172	C1ra	Complement Component 1, R Subcomponent A	–0.175069968	0.559598517
ENSMUSG00000016493	Cd46	Cd46 Antigen, Complement Regulatory Protein	–0.222483616	0.663841819
ENSMUSG00000042190	Cmklr1	Chemokine-Like Receptor 1	–0.253811222	0.169253436
ENSMUSG00000026399	Cd55	Cd55 Molecule, Decay Accelerating Factor For Complement	–0.292896576	0.067893029
ENSMUSG00000016481	Cr1l	Complement Component (3B/4B) Receptor 1-Like	–0.297021748	0.168692966
ENSMUSG00000016481	Cr1l	Complement Component (3B/4B) Receptor 1-Like	–0.297021748	0.168692966
ENSMUSG00000026938	Fcna	Ficolin A	–0.339976874	0.492867204
ENSMUSG00000023224	Serping1	Serine (Or Cysteine) Peptidase Inhibitor, Clade G, Member 1	–0.380740967	0.143743832
ENSMUSG00000061780	Cfd	Complement Factor D (Adipsin)	–0.519289828	0.110160899
ENSMUSG00000038527	C1rl	Complement Component 1, R Subcomponent-Like	–0.794072347	0.165929881
ENSMUSG00000026365	Cfh	Complement Component Factor H	–1.381019074	0.000280545
ENSMUSG00000079105	C7	Complement Component 7	–2.282311274	0.003259106

Pathway enrichment analysis revealed that two molecular networks identified as hallmarks of the 6 m state (blood vessel morphogenesis, and regulation of cell migration-both indicative of vessel inflammation) were also enriched in the 12 m state (Fig. 1B). Transcriptomics also defined a common signature between 6 m and 12 m BlAJ muscles composed of 10,638 genes that includes regulation of protein and cellular metabolic process, and signal transduction (Fig. 1B). Although several complement genes were differentially regulated in 6 m/12 m BlAJ muscles, the majority of them were included in the shared list of genes (Fig. 1C). Furthermore, gene set enrichment analysis (GSEA) revealed alterations of complement pathways in dysferlin-deficient mice (Fig.1D). Among disease-associated differentially expressed genes (DEGs) we found upregulation of several genes associated with inflammation, growth, and remodeling in 12 m BlAJ muscles. These included *Mitochondrial Translational Activator (Mss51)*, a key target of myostatin and TGF-β1 signaling involved in fatty acid oxidation and oxidative phosphorylation [47]; *stearoyl-CoA desaturase-2 (SCD2)*, which regulates lipid synthesis and oxidation [48, 49] and activation of monocytes [50]; *tenomodulin (Tnmd)*, a type II transmembrane glycoprotein functionally associated with angiogenesis inhibitor *Chm1* [51] and *Nuclear receptor subfamily 1 group D member 1* (*Nr1d1)*, whose expression regulates the infiltration of Mø [52, 53] (Table 1).

In order to validate these findings concerning inflammatory response, we performed western blot analysis on psoas muscle isolated from 12 m BlAJ and age-matched wild-type (WT) C57Bl mice. Expression of IP subunits PSMB8/PSMB9 and pentraxin 3 (PTX3)—a modulator of inflammation and innate immunity [54, 55]—was elevated in 12 m BlAJ skeletal muscle compared with those in age-matched controls (Fig. 1E). In addition, Toll-like receptor 4 (TLR4; central receptor of the innate immune system) and one of its inflammatory mediator—the tumor necrosis factor-α (TNFα)—were significantly upregulated in BlAJ mice compared to WT (Fig. 1E), thus confirming previous research in trained immunity of dysfelinopathy [56]. Interestingly, we found an increase of the ratio pP38/P38 and pAKT/AKT in 12 m BlAJ vs 12 m WT mice as previously recognized in several murine dystrophic animal models [57, 58]. Moreover, downregulation of the ratio pAMPKα/AMPKα was observed in 12 m BlAJ vs 12 m WT mice according to the literature describing the role of pAMPKα in plasma membrane repair of dysferlin-deficient myotubes [59]. Since the muscle microenvironment created by trained innate immune cells may have profound effects on T cell responses, such as altering the differentiation, polarization and function of T cell subtypes, we further characterized T cell repertoire through FACS analysis in 12 m BlAJ limb muscles. We did not observe significant modulation of the percentage of CD45+ cells and T-lymphocytes (Fig. 1F) but—interestingly—we determined an upregulation of CD4+ cells’ count (Supplementary Fig. 1A). In addition, we detected less naive CD4+ and CD8+ cells and increased percentage of effector CD4+ and CD8+ cells in affected dysferlin-deficient muscles (Fig. 1F–H). This suggests that altered innate cytokine production induced by trained immunity is likely to impact on T cell activation and fate in dysferlinopathy. Interestingly, CD4+ T-cells are often described in the perimysium of dysferlinopathic patients’ muscular biopsies [60].

**Fig. 2:**
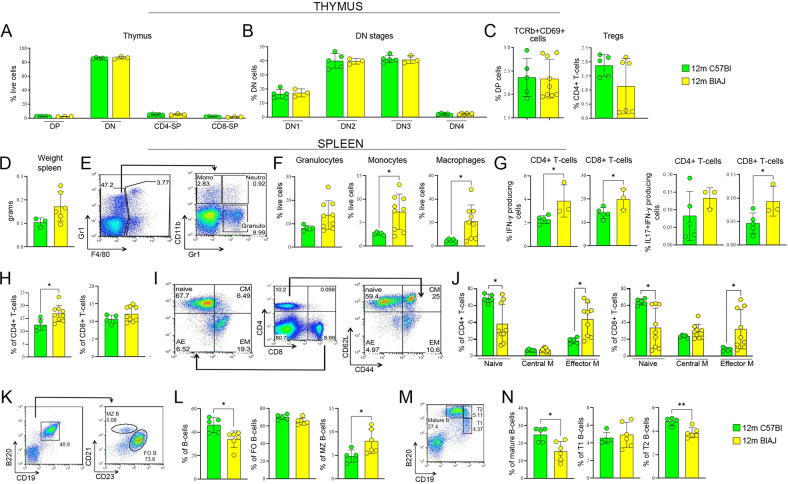
Evaluation of immune cells in dysferlinopathic mice. Representative FACS analysis of thymic subpopulations of 12 m C57Bl and 12 m BlAJ (**A**) and of DN stages (**B**). The amount of positively selected TCRβ+ CD69+ T cells and Tregs of 12 m C57Bl and 12 m BlAJ thymus (**C**). Spleen weight (in grams) in 12 m C57Bl and 12 m BlAJ mice (**D**). Representative FACS profile showing the percentage of each population of live cells: a gate of GR1-positive and F4/80 negative represents monocytes, neutrophils and granulocytes, further divided according to the expression of CD11b (**E**). Evaluation of spleen-derived granulocytes, monocytes and macrophages (**F**). FACS analysis of spleen-derived IFNγ- and IL17/IFNγ-producing CD4+ and CD8+ T cells (**G**) and of CD4+ and CD8+ T cells (**H**). Representative FACS profile is shown. The numbers within the panels indicate the percentage of each population of live cells: within the gate of CD4-positive or CD8-positive, two populations are separated by level of CD44 and CD62L expression (**I**). Evaluation of T-cell subsets—naive, central memory (CM), effector memory (EM)—expressing CD4 and/or CD8 in the spleens of 12 m C57Bl and 12 m BlAJ (**J**). Representative FACS profile showing the percentage of each population of live cells. Within the B220+ gate, two populations are separated by expression level of CD21 and CD23 (**K**). FACS analysis of total, follicular (FO, CD23+) and marginal zone (MZ, CD21+) splenic B cells of 12 m C57Bl and 12 m BlAJ mice (**L**). Representative FACS profile is shown. The numbers within the panels indicate the percentage of each population of live cells. Subpopulations are separated according to B220 and CD19 expression (**M**). FACS analysis of the percentage of mature B-cells (**M**) and evaluation of transitional T1 B (T1 B) and transitional T2 B (T2 B) splenic B cells of 12 m C57Bl and 12 m BlAJ mice (**N**). Data are presented as mean ± SD of *n* = 3 independent experiments with *n* = 3–9 animals/group. Student *t*-test: **p < 0.01.

The thymus is an essential organ for T cell development. The T cell progenitors enter the subcapsular cortical areas of the thymus as double negative (DN) for CD4 and CD8 expression, undergo V(D)J rearrangement of their T cell receptor (TCR) genes and become double positive (DP) CD4 and CD8 T cells [61]. To ensure self-tolerance, DP cells are subjected to negative selection by thymic epithelial cells presenting peptide self-antigens on their MHC class I and II molecules [62, 63]. To determine further whether muscle effector T cell of BlAJ might result from altered self-reactivity education mechanisms triggered by dysferlin-deficient thymus, we evaluated T cell maturation in thymus of 12 m BlAJ compared to age-matched C57Bl mice. No significant differences were observed in distribution of CD4-CD8- DN, CD4+CD8+ DP, CD4+CD8- and CD4-CD8+ single positive (SP) thymocytes (Fig. 2A) and double negative subpopulations—CD44+CD25- (DN1), CD44+CD25+ (DN2), CD44-CD25+ (DN3), and CD44-CD25- (DN4) (Fig. 2B). Similarly, no differences were found in the percentages of DP thymocytes upregulating TCR-α/β with concomitant expression of CD69, a feature of ongoing positive selection, and Tregs between BlAJ and WT mice (Fig. 2C). All these data indicate normal development of T cells within thymus and maintenance of central tolerance in 12 m BlAJ mice.

Then, we analyzed the immune system in the periphery. No differences in spleen weight were found between BlAJ and WT mice (Fig. 2D). However, we found increased numbers of splenic macrophages, while granulocytes and monocytes did not vary significantly between BlAJ and WT mice (Fig. 2E, F). Notably, the percentage of splenic activated IFNγ-producing CD4+/CD8+ T cells was higher in BlAJ vs WT (Fig. 2G). In agreement, 12 m BlAJ showed a significant increased amount of splenic effector CD4+ and CD8+ T-cells and central memory CD8+ T cells associated to reduced percentage of naive CD4+/CD8+ T cells (Fig. 2H, I). On the contrary, total percentages of B220+/CD19+ splenic B cells and frequencies of follicular (FO, CD23hi) and marginal zone (MZ, CD21hi) B cell subsets were similar between BlAJ and WT mice (Fig. 2J, K). Similarly, no significant variations into the amount of mature and transitional B cells were observed (Fig. 2L–N). All these findings demonstrated a distinct immune composition in dysferlin-deficient BlAJ mouse.

### Immunoproteasome inhibition drives M2 Mø polarization and reduces innate immunity in dysferlin-deficient mice

IP is crucially involved in innate immunity. We have previously shown that IP activity in the inflamed muscles promotes the production of pro-inflammatory cytokines in mdx murine model of Duchenne muscular dystrophy [41]. Having demonstrated that inflammatory mediators including IP subunits are upregulated in 12 m BlAJ mice, we next explored the effect of PSMB8 inhibitor ONX-0914.

**Fig. 3:**
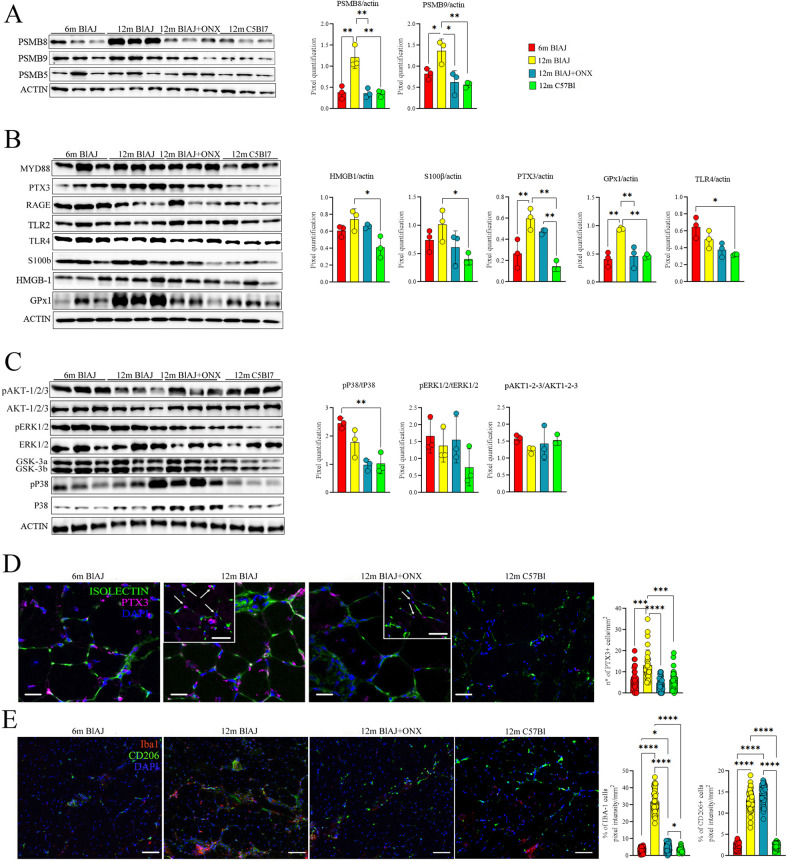
Proteomic and inflammatory features of dsyferlinopathic skeletal muscle mice are modulated following ONX-0914 treatment. Proteomic analysis of IP subunits (**A**); alarmins and inflammatory mediators (PTX3 and GPx1) (**B**); MAPK kinases and AKT1/2/3 and their phosphorylated isoforms (**C**) in psoas of 6 m BlAJ, 12 m BlAJ and BlAJ+ONX, 12 m C57Bl mice. Psoas stained with isolectin (in green), PTX3 (in magenta) with nuclei stained in DAPI (blu) (scale bar: 10 μm) and its magnification (scale bar: 10 μm) with white arrows indicating cells co-expressing PTX3 and isolectin. The histogram represents the counting of PTX3 fluorescence (**D**). Immunohistological staining of skeletal muscles for Iba1 (in red) and CD206 (in green). Nuclei were counterstained with DAPI (blue). Scale bar: 50 μm. Quantification of macrophage percentages. Mac1 were stained with Iba1+ and Mac2 with CD206+ in 6 m BIAJ, 12 m BlAJ and BlAJ+ONX, 12 m C57Bl skeletal muscles (**E**). Data are presented as mean ± SD of *n* = 3 independent experiments with *n* = 3–6 animals/group. One-way ANOVA, Tukey multiple comparison test for WB and non-parametric test followed by Kruskal–Wallis test for PTX3+ cells’ and macrophages’ counting: *p < 0.05, **p < 0.01, ***p < 0.001 and ****p < 0.0001.

Psoas muscle immunoblot analysis confirmed downregulation of PSMB8 and PSMB9 IP subunits in 12 m BlAJ mice treated with ONX-0914 (12 m BlAJ+ONX) (Fig. 3A). Among muscle inflammatory mediators, we found significant downregulation of the alarmins HMGB1, S100β, PTX3 and glutathione peroxidase-1 (GPx1) in 12 m BlAJ+ONX (Fig. 3B) while MYD88, RAGE and TLR2/TLR4 were similar between 12 m BlAJ and 12 m BlAJ+ONX (Supplementary Fig. 3B). The ratio pP38/P38 was restored toward WT levels while no differences of the ratio pERK1/2/ERK1/2 and pAKT/AKT were found between untreated and ONX-treated 12 m BlAJ. Similarly, no significant alterations were detected for GSK-3α and 3β expression (Fig. 3C and Supplementary Fig. 3B). In line with immunoblot evidence, we found that the number of perivascular PTX3+ inflammatory cells was dramatically decreased in 12 m BlAJ+ONX (Fig. 3D). This coincided with reduced pro-inflammatory Iba+CD206- M1 Mø and increased Iba1+ CD206+ M2 Mø in 12 m BlAJ+ONX (Fig. 3E).

To investigate whether IP activity of dysferlin-deficient Mø is of any functional relevance for macrophage polarization, we polarized monocytes isolated from 12 m BlAJ mice by treatment with TNFα.

We observed typical morphologies displayed by M1 Mø (rounded, “fried egg shape”) in the presence of TNFα. Interestingly, ONX-0914 induced elongated and irregularly shaped M2 polarized Mø (Supplementary Fig. 4A). Immunofluorescence analysis confirmed the co-expression of Iba1 (Mø marker) and CD206 (typical M2 marker) only in the ONX-0914 treated dysferlin-deficient Mø (Supplementary Fig. 4A). Western Blotting analysis of protein samples extracted from BlAJ Mø cultures showed that ONX-0914 had a direct impact on the downregulation of IP subunits, TNFα/TLR4/AKT pathway, mitochondrial phosphorylation, autophagy and C3aR1/C5aR1 downstream pathways (Supplementary Fig. 3B). Thus, these data corroborate evidences that IP inhibition in dysferlin-deficient macrophages induces a differential signaling response to TNFα skewing the macrophage polarization toward the M2 phenotype.

**Fig. 4:**
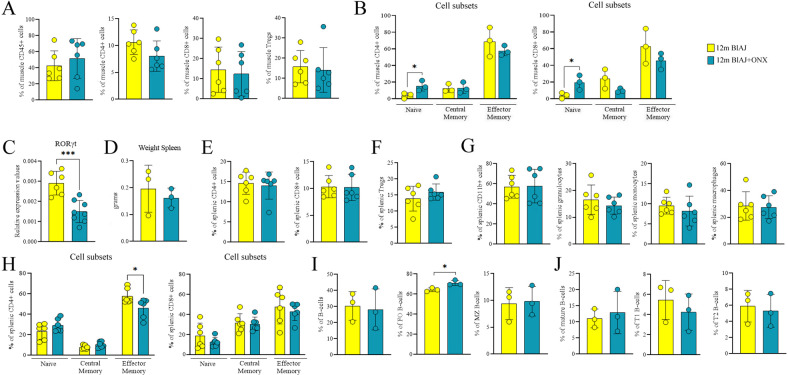
Inflammatory cells of dsyferlinopathic mice are modulated by ONX-0914 treatment. FACS analysis of CD45+, CD4+/CD8+ T-lymphocytes and Tregs infiltrating the 12 m BlAJ and 12 m BlAJ+ONX skeletal muscles (**A**). Evaluation of muscle-derived CD4+ and CD8+ T-cell subsets (**B**). RT-qPCR analysis of Th17-dependent ROR-γt in 12 m BlAJ and 12 m BlAJ+ONX skeletal muscles (**C**). Determination of splenic weight in 12 m BlAJ and 12 m BlAJ+ONX mice (**D**). FACS analysis assessing the percentage of splenic CD4+/CD8+ T cell subsets (**E**) and FoxP3+ CD25+ Tregs (**F**) of 12 m BlAJ and 12 m BlAJ+ONX mice. Percentage of macrophages, neutrophils, monocytes and granulocytes (**G**) and naive, central memory and effector memory CD4+ and CD8+ cells (**H**) in the spleen of 12 m BlAJ and 12 m BlAJ+ONX mice. Evaluation of B-cell counts (**I**) and B-cell subsets (**J**) of 12 m BlAJ and 12 m BlAJ+ONX mice. Data are presented as mean ± SD of *n* = 3 independent experiments with *n* = 6 animals/group (and technical replicates for RT-qPCR). Student *t*-test: *p < 0.05 and ***p < 0.001.

As IP subunits are involved in the activation of CD8+ T cell response, we further characterized T cell repertoire in 12 m BlAJ+ONX. Cytofluorimetric analysis of psoas muscles of 12 m BlAJ+ONX showed a significantly reduced number of infiltrating CD4+ and CD8+ T cells (Fig. 4A and Supplementary Fig. 2A) and increased percentage of naive CD4+ and CD8+ T cells (Fig. 4B). Muscle Tregs percentage was unaffected by ONX-0914 treatment (Fig. 4A). RT-qPCR experiments evidenced reduced expression of *ROR*γ*t*, indicating diminished Th17 pro-inflammatory T cells (Fig. 4C). However, 12 m BlAJ+ONX mice did not modify cellularity and weight of spleen (Fig. 4D) neither the amount of CD4+/CD8+ lymphocytes (Fig. 4E) nor Tregs (Fig. 4F), whereas a slight—but not significant—reduction of spleen amounts of neutrophils, macrophages, monocytes and granulocytes was found (Fig. 4G). Interestingly spleen of 12 m BlAJ+ONX mice showed a significant downregulation of CD4+ effector T-cells, reflecting a reduced T cell activation state (Fig. 4H). Consistent with previous evidences of proteasome inhibition affecting B cell maturation [64, 65], we found significant increase of FO B cells (Fig. 4I) while mature B-cells were not affected by ONX-0914 (Fig. 4J). To further explore changes in the immune repertoire of ONX-0914-treated psoas muscle we performed RNA sequencing in 12 m BlAJ+ONX-0914 compared to 12 m BlAJ muscles (Supplementary Table 1). We observed that 14 disease-associated DEGs were restored toward unaffected 6 m BlAJ levels including three upregulated (*Krt18, Slc23a3* and *Etfb)* and 11 downregulated (*Cd209a, BMI-1, Bcl, Gmfb, Cdkn1c, Ripor2, Ermp1, PCNP* and *Zfp397*) genes (Supplementary Fig. 5A). These ONX-rescued genes are associated to pathways involved in multiple cellular processes such as cell growth, oxidative stress and metabolism (e.g. Wnt, AKT, Notch, Hedgehog and receptor tyrosine kinase pathways), macrophages phagocytosis, proteasome-mediated catabolic process and protein post-translational modifications [66, 67]. We likewise observed that although genes belonging to both T-cell and macrophage GO pathways are differentially expressed between pre-symptomatic 6 m and affected 12 m BlAJ mice, only genes linked to macrophage function were statistically differentially expressed after ONX-0914 administration (Supplementary Fig. 5B, C). These findings suggest that both macrophage and T-cell-mediated muscle immunity contributes to dysferlinopathy whereas ONX-0914 treatment exert its effects more restrictedly on macrophages population.

### Immunoproteasome inhibition induces vessel remodeling in muscle of dysferlin-deficient mice

**Fig. 5:**
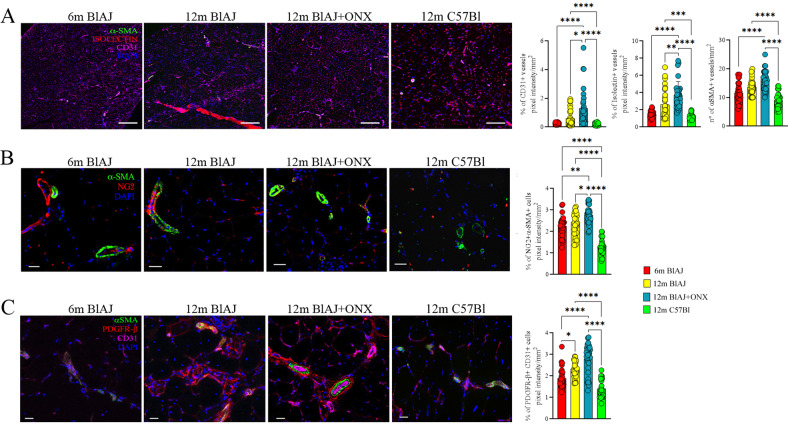
ONX-0914 treatment affects angiogenesis in dsyferlinopathic mice. Representative images and quantification of αSMA (in green), isolectin (in red) and CD31 (in magenta) staining of psoas muscles from 6 m BlAJ, 12 m BlAJ and BlAJ+ONX, 12 m C57Bl. Nuclei were stained in DAPI (blue) (scale bar: 200 μm) (**A**). Psoas of 6 m BlAJ, 12 m BlAJ and BlAJ+ONX, 12 m C57Bl mice stained with αSMA (in green) and NG2 (in red) with nuclei stained in DAPI (blu) (scale bar: 25 μm) and their quantification (**B**). Psoas of 6 m BlAJ, 12 m BlAJ and BlAJ+ONX, 12 m C57Bl mice stained with αSMA (in green), PDGFRβ+ (in red) and CD31 (in magenta) with nuclei stained in DAPI (blu) (scale bar: 50 and 25 μm) and their quantification (**C**). Data are presented as mean ± SD of *n* = 3 independent experiments with *n* = 3–6 animals/group. One-way ANOVA and non-parametric test followed by Kruskal–Wallis test: *p < 0.05, **p < 0.01, ***p < 0.001 and ****p < 0.0001.

**Fig. 6:**
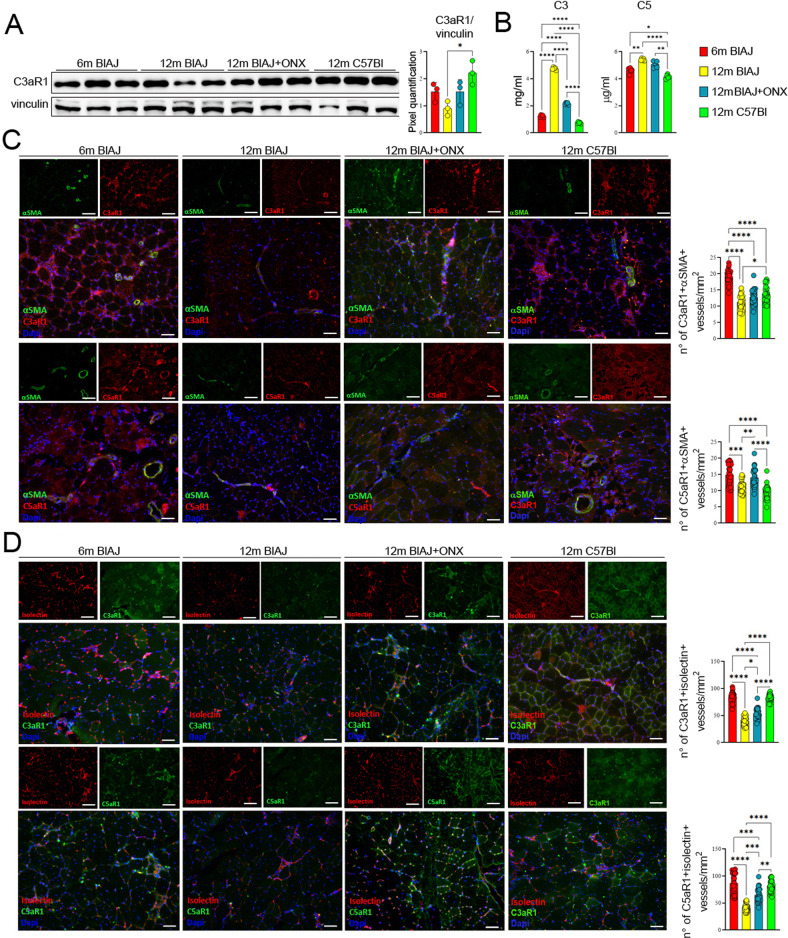
Complement-cascade proteins are co-expressed with endothelial cell markers in ONX-0914-treated mice. Proteomic analysis of C3aR1 in psoas of 6 m BlAJ, 12 m BlAJ and BlAJ+ONX, 12 m C57Bl mice (**A**). Evaluation of C3 and C5 concentration in the serum of 6 m BlAJ, 12 m BlAJ and BlAJ+ONX, 12 m C57Bl mice (**B**). Representative images and quantification of psoas muscles from 6 m BlAJ, 12 m BlAJ and BlAJ+ONX, 12 m C57Bl with big vessels co-stained with αSMA (in green) and C3aR1/CD88/CD93 (in red). Nuclei were stained in DAPI (blue) (scale bar: 100 μm) (**C**). Representative images and quantification of psoas muscles from 6 m BlAJ, 12 m BlAJ and BlAJ+ONX, 12 m C57Bl with small vessels co-stained with isolectin (in green) and C3aR1/CD88/CD93 (in red). Nuclei were stained in DAPI (blue) (scale bar: 50 μm) (**D**). Data are presented as mean ± SD of *n* = 3 independent experiments with *n* = 3–6 animals/group. One-way ANOVA, Tukey multiple comparison test for WB and non-parametric test followed by Kruskal–Wallis test for vessels’ count: *p < 0.05, **p < 0.01, ***p < 0.001 and ****p < 0.0001.

We further sought to investigate whether sustained inflammation in dysferlin-deficient BlAJ muscle might be in part driven by altered vessels. The examination of the dysferlin-deficient muscle microvasculature as a precondition for the infiltration of immune cells showed that the endothelial layer of asymptomatic 6 m BlAJ muscle is characterized by tight intercellular junctions (Fig. 5A–C). Oppositely, due to high pro-inflammatory signaling, the network of blood vessels of 12 m BlAJ was chaotic, low in NG2+ pericyte coverage and had loose inter-endothelial cell junctions generating leaky vessels (Fig. 5A–C). Staining of serial psoas muscle sections of BlAJ demonstrated that ONX-0914 efficiently increased the number of CD31+ capillaries and small arterioles co-expressing isolectin and α-smooth muscle actin (αSMA) in 12 m BlAJ+ONX (Fig. 5A). Importantly, we found a significant increase of the double positive NG2+ αSMA+ pericytes which correctly covered capillaries and small arteries of 12 m BlAJ+ONX mice (Fig. 5B). Immunostaining of arterioles revealed that αSMA+ cells are located in the arterial adventitia distinct from CD31+ endothelial cells in 12 m BlAJ+ONX, whereas untreated 12 m BlAJ muscles showed loss of vessel integrity with αSMA+ cells prevalently located outside vascular wall, suggesting reduction of their contractile function (Fig. 5C). Notably, the number of double positive CD31+ PDGFRβ+ hematopoietic/endothelial precursors, which drive endothelial cells differentiation [68], was significantly increased in 12 m BlAJ+ONX mice (Fig. 5C). Both C3a and C5a have been described to mediate vasodilation, increase vascular permeability, chemotaxis, and cytokine production by innate immune cells through interaction with their C3aR1 and C5aR1 specific receptors [43, 69]. Muscle expression of C3aR1 and C5aR1 was mainly observed in isolectin+ capillaries and αSMA+ arterioles of asymptomatic 6 m and 12 m BlAJ+ONX mice (Fig. 6A–C). Similarly, WB analysis of C3aR1 showed comparable expression between 6 m BlAJ and 12 m BlAJ+ONX mice (Fig. 6A). Since complement pathways contribute to disease progression in dysferlinopathy [20], C3 and C5 serum levels were evaluated in BlAJ at 6 and 12 months vs 12 m BlAJ+ONX. Interestingly, we found a significant decrease of C3 serum levels in 12 m BlAJ+ONX compared to 12 m BlAJ (Fig. 6B). All these data suggest that IP inhibition modulates central complement component C3 rather of terminal C5 activation and restored vessel stability through C3aR1 and C5aR1 vessel expression.

### Dysferlinopathy in BlAJ mice is ameliorated by ONX-0914 treatment

**Fig. 7:**
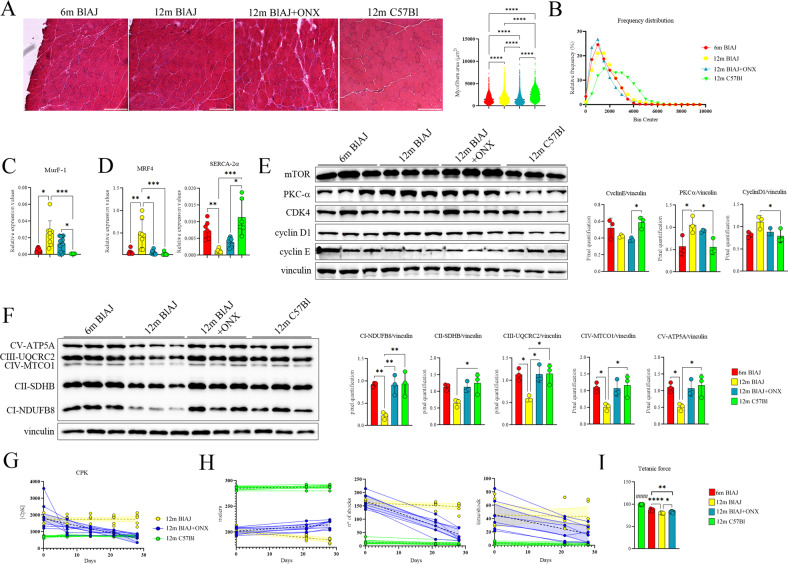
Immunoproteasome inhibition ameliorates muscle performance of dsyferlin-deficient 12 m BlAJ mice. Representative EE staining and quantification of myofiber area (**A**) and relative frequency of the myofiber cross-sectional area (CSA) expressed as the frequency distribution (**B**) of psoas of 6 m BlAJ, 12 m BlAJ and BlAJ+ONX, 12 m C57Bl mice. For morphometric analysis, images were quantified with ImageJ software for each mouse (scale bar: 200 μm). RT-qPCR analysis showed upregulation of *MuRF-1* expression in 12 BlAJ muscles related to 12 m BlAJ+ONX and 6 m BlAJ (**C**). RT-qPCR analysis showed higher expression of *MRF-4* and lower amount of *SERCA2*α in muscles of 12 m BlAJ mice (**D**). WB analysis of mTOR and other proteins regulating cell-cycle progression (**E**); OXPHOS (**F**) in psoas of 6 m BlAJ, 12 m BlAJ and BlAJ+ONX, 12 m C57Bl mice. Downregulation of CpK concentration following ONX-0914 treatment in BlAJ mice (**G**). Treadmill test determined amelioration of walking distance, time/shock and numbers of shock in 12 m BlAJ+ONX mice related to untreated ones (**H**). Significant improvement of tetanic muscular force was found in 12 m BlAJ+ONX related to untreated 12 m BlAJ (**I**). Data are presented as mean ± SD of *n* = 3 independent experiments with *n* = 3–6 animals/group (and technical replicates for RT-qPCR). One-way ANOVA, Tukey multiple comparison test for WB and non-parametric test followed by Kruskal–Wallis test for myofibers’ area and tetanic muscle force: *p < 0.05, **p < 0.01, and ****p < 0.0001. For tetanic muscle force measurement, the values of 12 m C57Bl were significant upregulated related to all the other mice with *p* < 0.0001 and indicated in the graph with ####.

**Fig. 8:**
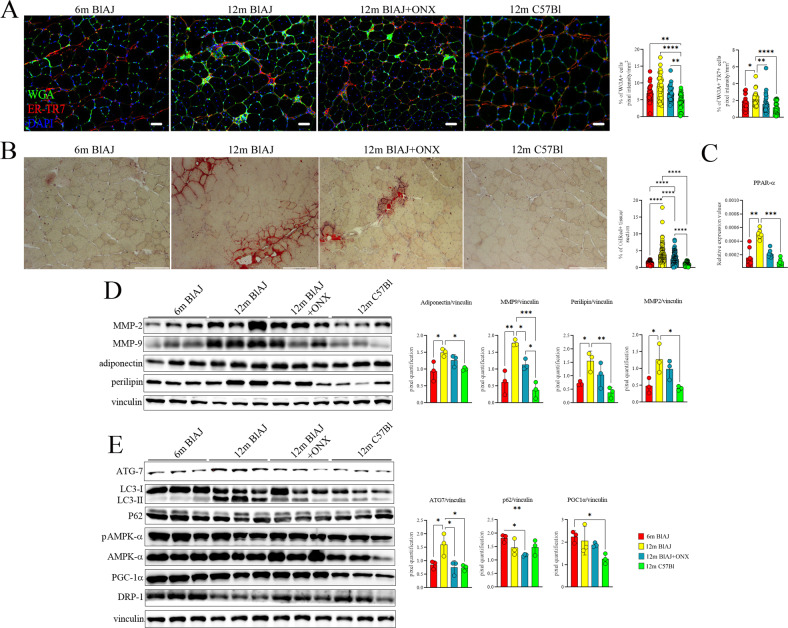
Immunoproteasome inhibition reduces fibro-adipogenesis of dsyferlin-deficient 12 m BlAJ mice. Fluorescent staining of WGA+ (in green) and TR7+ (in red) while nuclei are in DAPI (blu) (scale bar: 50 μm) in psoas of 6 m BlAJ, 12 m BlAJ and BlAJ+ONX, 12 m C57Bl mice (**A**). ORO staining in psoas of 6 m BlAJ, 12 m BlAJ and BlAJ+ONX, 12 m C57Bl mice (**B**). Expression of pro-adipogenic mediators as *PPAR*α (**C**)—in RT-qPCR—and perilipin, adiponectin and metalloproteinases (**D**)—in WB—was downregulated following ONX-0914 treatment. Proteomic analysis of autophagy mediators, DRP1, and PGC1α (**E**). Data are presented as mean ± SD of *n* = 3 independent experiments with *n* = 3–6 animals/group (and technical replicates for RT-qPCR). One-way ANOVA, Tukey multiple comparison test for WB and non-parametric test followed by Kruskal–Wallis test for WGA+/TR7+ cells’ counting, Oil Red evaluation and RT-qPCR: *p < 0.05, **p < 0.01, ***p < 0.001 and ****p < 0.0001.

Next, we investigated whether the modification of Mø and T cell response induced by ONX-0914 might impact on disease severity of 12 m BlAJ mice. Histological analysis of psoas muscle demonstrated that 12 m BlAJ mice had higher myofibers area than asymptomatic 6 m mice. Moreover, 12 m BlAJ+ONX showed significant reduction of myofibers area compared to 6 m and 12 m BlAJ mice (Fig. 7A). Frequency distribution analysis confirmed the smaller area of psoas myofibers in 12 m BlAJ+ONX related to both 6 m and 12 m BlAJ mice (25% percentile: 807 for 6 m BlAJ, 976 for 12 m BlAJ and 706 for 12 m BlAJ+ONX; 75% percentile: 2087 for 6 m BlAJ; 2303 for 12 m BlAJ and 1864 for 12 m BlAJ+ONX) (Fig. 7B). Downregulation of *MuRF-1* in 12 m BlAJ+ONX vs 12 m BlAJ muscles (Fig. 7C) excluded that reduction of myofiber area and frequency distribution correspond to muscle atrophy. These observations suggest that ONX-0914 treatment induces muscle regeneration in affected 12 m BlAJ muscle. According to these data, we found significant decrease of *MRF-4* and opposite significant increase of *SERCA2a* in 12 m BlAJ+ONX vs 12 m BlAJ (Fig. 7D). Although not significant, we observed a trend of downregulation of PKCα and Cyclin E and upregulation of mTOR in 12 m BlAJ+ONX (Fig. 7E and Supplementary Fig. 3C) whereas other inflammatory mediators were not modified (Supplementary Fig. 3C). We thus evaluated the expression pattern of genes involved in fatty acid and glucose metabolism pathways and observed downregulation of pyruvate dehydrogenase kinase-4 (*pdk4*) and of medium-chain acyl-CoA dehydrogenase (*mcad*) in 12 m BlAJ+ONX vs 12 m BlAJ, whereas no modifications of glucose transporter 4 (*Glut4*), glycolytic enzymes as pyruvate dehydrogenase (*pdh*) and phosphor-fructokinase (*pfk*) were detected (Supplementary Fig. 3D). In addition, we found significant differences in mitochondrial metabolism by the expression of C2, C3 and C4 respiratory chain subunits, whose values in 12 m BlAJ+ONX mice appeared similar to those of 6 m BlAJ (Fig. 7F). These observations relayed to a tendency of PGC1α—that mediates mitochondrial biogenesis [70, 71]—since its expression was downregulated in 12 m BlAJ, returning to pre-symptomatic levels following ONX-0914 treatment (Fig. 8E and Supplementary Fig. 3C) and correlate with the modulation of *Mss51* (Table 1A, upregulated genes in 12 m BlAJ). No differences were found in the expression of dynamin-related protein, DRP1—normally involved in mitochondrial division in mammalian cells [72] (Supplementary Fig. 3C). Since fibro-adipogenic alterations were described in BlAJ mice [73], we extended our analysis testing muscular modulation by Wheat Germ Agglutinin (WGA)/fibroblast ER-TR7 (TR7) markers. Interestingly, while increased WGA/TR7 staining was detected in 12 m BlAJ mice, these fibro-adipogenic markers were significantly reduced upon ONX treatment (Fig. 8A), suggesting that inflammatory and metabolic modulation induced by ONX-0914 determines amelioration of the fibro-adipogenic replacement in dysferlinopathic muscle. In line, we found significant decrease of neutral lipid stain Oil Red O following ONX-0914 treatment (Fig. 8B), and significant downregulation of *PPAR*α, adiponectin and perilipin in 12 m BlAJ+ONX vs 12 m BlAJ mice, according to *SCD2* expression (Fig. 8C, D). Consistently, ONX-0914 treatment reduced the expression of MMP2 and—more significantly—MMP9 (Fig. 8D). Since different evidences described the role of fibro-adipogenic progenitors in controlling autophagy [74, 75] and, based on the above mentioned findings, we investigated the expression of ATG-7, microtubule-associated protein 1 and 2 light chain 3 (LC3-I and LC3-II) and of LC3-binding chaperone P62. Interestingly, we found activated autophagic flux featured by increased ratio LC3-II/LCR-I and downregulation of ATG-7 and P62 expression in 12 m BlAJ+ONX related to untreated BlAJ mice (Fig. 8E). Another candidate in autophagy regulation is AMPKα because it senses metabolic modifications to maintain cellular energy homeostasis [76]. Although not significant, muscle tissues of ONX-treated 12 m BlAJ showed an increased trend of the ratio of pAMPKα/AMPKα compared to untreated 6 m and 12 m BlAJ (Fig. 8E), whereas the ratio pP38/P38 was restored toward WT levels (Fig. 3C). No differences of the ratio pAKT/AKT were found between untreated and ONX-treated 12 m BlAJ (Fig. 3C).

To assess dysferlinopathic muscle damage, we measured the levels of serum creatine phospho-kinases (CpK). Compared to 12 m BlAJ muscles, there was a significant decrease of CpK levels in 12 m BlAJ+ONX, similar to levels of 12 m C57Bl mice (Fig. 7G), indicating a link between reduced inflammatory and fibro-adipogenic features and muscle integrity of dysferlinopathic ONX-0914-treated muscles. Consistent with morphological and metabolic finding, we examined whether ONX-0914 treatment contributes to muscle performance amelioration in dysferlinopathy. Notably, treadmill tests of 12 m BlAJ+ONX showed increased run distance, reduced time/shock and significant reduction in the number of shocks (Fig. 7H).

Indeed, tetanic force of TA muscle of 12 m BlAJ+ONX was significantly increased related to age-matched BlAJ mice (Fig. 7I). Hence, these findings propose the IP to be an interesting therapeutic target for the treatment of dysferlinopathy.

## Discussion

Collectively, our data show that disruption of the immunoproteasome system mitigates muscle pathology in dysferlin-deficient mice. Dysferlin deficiency in myofibers inhibits sarcolemmal repair [77], disrupts calcium homeostasis at the T-tubules [78] and alters immune response [79] leading to progressive and debilitating muscular disorders characterized by extremely low regenerative capacity, inflammatory infiltrates and fibro-adipogenesis. It is still not clear why dysferlinopathy shows such a broad age of onset and clinical spectrum and such extensive muscle inflammation. In recent years, the work of Haynes showed that the lack of dysferlin leads to pathological modulation of fatty acids metabolism both in skeletal muscle and adipose tissue [57]. Indeed, Baek et al. determined that dysferlinopathic muscles recruited larger amount of inflammatory M1 macrophages, favoring their proliferation and conveying muscle fibers more prone to necrosis and apoptosis [44].

T lymphocytes activation [56] and complement-mediated inflammation [20, 80] allow the secretion of pro-inflammatory molecules that render the endothelial wall permeable to inflammatory macrophages and neutrophils, suggesting a link between innate immunity and disease onset/progression of dysferlinopathy. In this sense, blockade of innate immune activation [56]—and in particular of the cells that provoke upregulation of inflammosome and NF-kB pathway—might represent an innovative approach preventing myofibers loss of dysferlinopathic muscle. IP subunits are expressed in immune cells and in an inflammatory environment, and therefore, selective IP inhibitors can be applied to diminish inflammatory features of muscular dystrophy [23, 40, 41]. Here, we provide evidence that immune response in dysferlin-deficient BlAJ mice is driven by T cell and Mø activation. Nevertheless, muscles from BlAJ mouse show a greater abundance of PSMB8 and PSMB9 IP subunits. The PSMB8 inhibitor ONX-0914 contributes to reduce the number of splenic and muscular CD4+/CD8+-effector T cells. Accordingly, ONX-0914 treatment targets macrophages polarization increasing the number of M2 Mø that are required for efficient muscle regeneration. Another effect of IP inhibition was the restoration of glucose and mitochondrial metabolism in affected 12 m dysferlin-deficient mice. This is consistent with modifications of AMPKα/p38 MAPK/mTOR signaling pathway. Furthermore, the metabolic reprogramming that we observed in 12 m BlAJ+ONX correlates with decreased adipogenesis, and increased autophagic flux, therefore explaining the amelioration in the extracellular matrix structure and the rescue of muscle performance.

Indeed, we described significant ONX-0914 modulation of C3aR1/C5aR1 inducing Mø polarization in bone marrow-derived macrophages of 12 m BlAJ mice. Complement C3a–C3aR1, but not C5a–C5aR1, signaling is critical for recruitment of circulating monocytes into damaged muscle where they promoted muscle regeneration [15]. Moreover, the activation of the central component C3 was found to accelerate muscle injury in dysferlin-null mice on the C57Bl inbred background [20]. Of note, ONX-0914 treatment induced a significant decrease of complement C3 serum levels in 12 m BlAJ+ONX. Recently, a substantial muscle deposition of complement C5b-9 with a predominance of macrophages was described as the unique inflammatory pattern of human dysferlinopathies [81]. Bioinformatics analysis of RNA-seq experiments showed that complement genes were enriched in 6 m BlAJ muscle associated with complement receptors downregulation in 12 m BlAJ muscle. This finding indicated that complement signaling might be important for inflammatory cell recruitment and timing of phenotypic M1/M2 Mø transition thus promoting either muscle inflammation or repair depending upon the stage of dysferlinopathy. We identified cellular/molecular mechanisms that can potentially link complement and dysferlin-deficient macrophages. C3aR1 activation could increase the phosphorylation of AKT and p38 as a downstream signaling pathway, which was reported to promote the transcription of HMGB1 and PTX3 [82]. Moreover, several lines of evidence supports that complement modulates innate immunity players to regulate angiogenesis [43] and pericyte growth [83].

Given that dysferlin-deficient muscle vessels showed loss of wall integrity, we speculated that immune response started from activated inflamed vessels. Consistent with this we show significant C3aR1 and C5aR1 accumulation in capillaries and small arterioles of 6 m BlAJ vs 12 BlAJ muscles. Use of ONX-0914 determines rescue of vessel integrity and increase of NG2+ pericyte coverage. Moreover, ONX-0914 upregulated the expression of C3aR1 and C5aR1 on muscle vessels of 12 BlAJ mice. The relationship between C3aR1 and C5aR1 and vessel remains obscure and is not yet reported in muscular dystrophies. Emerging evidence suggests that C3aR confers vessel protection through C3a/C3aR axis–mediated negative regulation of pro-inflammatory responses and modulation of macrophage toward the anti-inflammatory phenotype [16]. One possible explanation is that C3aR1 and C5aR1 might have a protective role in muscle vessels that is insufficiently controlled in inflamed muscles. In this sense, PTX3—mainly induced by pro-inflammatory cytokines—was found prevalently expressed in capillaries and small arterioles of 12 m BlAJ muscles and its expression downregulated in ONX-0914 treated muscles to levels toward 6 m BlAJ mice. Given that PTX3 impairs the vascular regenerative response [84, 85] modulating vascular inflammation [86], it is reasonable to assume that PTX3 could be partly responsible for endothelial complement modulation in dysferlin-deficient muscles. Therefore, our findings suggest that IP inhibition modulates C3 serum levels and C3aR1/C5aR1 expression in Mø toward an anti-inflammatory M2 phenotype, leading to Mø-mediated vessel stability in dysferlin-deficient mice. These data are in agreement with previous evidences indicating that both complement and Mø interact closely to maintain process of angiogenesis [42, 43]. Moreover, modulation of innate immunity induced by ONX-0914 determines metabolic modifications that could also influence endothelial-pericyte interactions [87]. In particular, *Pdk4* and *mcad* over-expression associated to M1 Mø polarization [88–90] and loss of small vessels [91] are rescued by ONX-0914 treatment in 12 m BlAJ muscle tissue.

Dysferlinopathy is often associated with weakness, decreased muscle regeneration and increased fibro-adipogenesis. We show that IP inhibition by ONX-0914 increases the number of regenerating myofibers and reduces fibro-adipogenesis of dysferlinopathic muscle of BlAJ mice. Importantly, we provide evidence of decrease of damaged alizarin red positive myofibers that was associated to a significant decrease of CpK levels in ONX-0914 treated 12 m BlAJ mice. Finally, mechanical analysis of intact muscles revealed that muscle force was strongly increased in ONX-0914 treated 12 m BlAJ mice and these data correlated to increased muscle performance.

In summary, we provide the first evidence of IP subunits over-expression in affected dysferlin-deficient 12 m BlAJ muscles as a central target to prevent T cell and macrophage immune response, ameliorate vascular architecture defects and improve muscle force. We suggest that increasing our understanding of the contribution of aberrant IP expression to the pathophysiology of dysferlinopathy will ultimately lead to the development of novel therapeutic approaches. Even if side effects associated with their long-term use are not completely avoidable, the use of IP inhibitors could represent an encouraging starting point for the development of new immunotherapy for dysferlinopathy.

## Materials and methods

### Ethic statement

The research procedures described were approved by the ethics committee of the University of Milan (CR937-G). This study was performed in accordance with International Conference on Harmonisation of Good Clinical Practice guidelines, the Declaration of Helsinki (2008) and the European Directive 2001/20/EC. Procedures involving living animals were approved by local ethics committees, conforming to Italian law (D.L.vo 116/92 and subsequent additions). This work was authorized by the Ministry of Health and Local University of Milan Committee with the protocol authorization numbers 10/10-2009/2010 and 6/13-2012/2013. Twelve-month-old normal (12 m C57Bl), 6-month-old and 12-month-old dysferlinophatic (6 m BlAJ and 12 m BlAJ) mice were provided by Charles River (Calco, Lecco, Italy) and caged with comfort and safety, in controlled ambient (12-h light, 12-h dark) at a temperature between 2 and 24 °C. The mice had free access to clean water and food. The immunoproteasome inhibitor ONX-0914 (Clini Sciences, France, 6 mg/Kg) was injected intraperitoneally into 12 m BlAJ for 5 weeks (two injections per week, *n* = 10). Untreated age-matched BlAJ mice were used as control. Mice were sacrificed by cervical dislocation according to the Italian country Law. Randomization within blocks was performed to allocate the animals to different experimental procedures. To avoid that the effects of our treatments on mice had been overestimated thus diminishing the reliability of our results, the laboratory members that analyzed the mice were blinded regarding the treatment(s) that animals received, during all the experimental procedures. Animals that eventually suffered from clinical complications during each treatment (enhancement of stress, motor impairments) were excluded from the experimental plan.

### RNA-seq analysis

To prepare the library and sequence the DNA, 150–300 ng of total RNA from muscles of 6 m and 12 m BlAJ and 12 m BlAJ+ONX mice was determined by InvitrogenTM QubitTM; high-sensitivity spectrofluorometric measurement was poly-A selected and reverse transcribed using Illumina’s TruSeq stranded mRNA library preparation kit. Each sample was fitted with one of 96 adapters containing a different 8 base molecular barcode for high level multiplexing and libraries were sequenced on an Illumina NovaSeqTM 6000. To ensure the quality of the experiment, the FASTQ were checked through FastQC as in [92]. We determined transcript/gene abundance with kallisto3 [93] and we used a specific transcriptome index to quantify transcript abundance [94]. GraphPad Prism (release 9.0.2) was used for volcano plot analysis of RNA-seq expression data. Differentially expressed genes were identified by *p* value < 0.05. Genes related to complement function were identified via the Gene Ontology Browser by Mouse Genome Informatics. The following pathways were selected: complement activation (Biological process; GO: 0006956) and complement receptor activity (Molecular function; GO: 000487). PANTHER (release 17.0) Gene List Analysis tool was used to determine Gene ontology (GO) enrichment [95] after identification of differentially and non-differentially expressed genes among 6 m and 12 m BlAJ mice. GSEA was performed *via* dedicated software (release 4.2.3) by Molecular Signatures Database (MSigDB). The “Hallmark_Complement” annotated gene set (included in the “Hallmark” gene set collection v7.5.1) was used for analysis of ranked gene lists.

### Cytofluorimetric analysis of cells from murine thymus, spleen and muscles

Muscles, thymus and spleen were removed from treated and untreated 12 m mice to determine the amount of different immune subpopulations by FACS analysis. Muscles were excised and extensively washed in PBS to removed blood contaminants [96], cut in small pieces and digested for 1 h with liberase 0.2 mg/ml (Invitrogen). Cells derived from tissue dissociation were then filtered with 70 μm mesh filters while undigested tissues were mashed with a plunger through the filters. Cell suspensions were multiple-labeled with different combinations of antibodies to recognize specific subpopulations. Spleens were coarsely cut with a scalpel in small pieces and smashed through a 70 μm mesh filters onto a petri dish using a 3-ml syringe plunger. Red blood cells were lysed by adding 2 ml of ACK Lysis buffer, purchased by Thermo Fisher Scientific. Cells were incubated for 15 min and reaction was blocked by adding 2 ml HBSS (GIBCO) with 10% FBS. Cells were collected, transferred in 15 ml conical tubes and centrifuged before labeling for FACS. For the examination of thymus cellularity, thymi were depleted from fat and connective tissue, transferred to 6-well plate containing Liberase (Invitrogen) solution, incubated at 37 °C for 20 min and subsequently dissociated as described in details by [97]. For muscle: CD45 PerCp; CD4 PeCy7; CD8 efluor 450; CD44 FITC; CD62L PE; CD25 APC; B220 APC-Cy7. CD45 PerCp; CD4 Pacific Blue; CD25 APC; GITR PeCy7; CD3 FITC; B220 APC-Cy7. For spleen: CD4 PeCy7; CD8 efluor 450; CD44 FITC; CD62L PE. CD4 Pacific Blue; CD8 APC-Cy7; Foxp3 Alexa fluor 488; CD25 APC; CTLA4 PE; ICOS PerCP. CD19 PerCp; B220 APC-Cy7; CD21 Pacific Blue; CD23 PeCy7; IgD PE; IgM APC. Gr1 APC-Cy7; F4/80 PeCy7; CD11b PE; CD11c FITC. CD4 PAcific Blue; CD8 APC-Cy7; IL17 PE; IFNγ APC; Foxp3 Alexa Fluor 488. For thymus: CD4 Pacific Blue; CD8 APC-Cy7; CD44 FITC; CD25 APC. CD4 Pacific Blue; CD8 APC-Cy7; CD69 FITC; TCRβ PE; Qa2 APC. CD4 Pacific Blue; CD8 APC-Cy7; CD25 APC; Foxp3 Alexa fluor 488. All the antibodies were purchased from eBioscience (San Diego), except for CD44 FITC, CD45 PerCp, IgD PE, and TCRβ PE obtained from BD (New Jersey) and CD4 Pacific Blue from BioLegend (San Diego, CA).

Cells were isolated with A-FACS Aria machine (BD Bioscience, New Jersey); data were acquired with the Cytomics FC500 (Beckman-Coulter) machine and analyzed with CXP 2.1 software

### WB analysis

Total proteins were obtained from skeletal muscles isolated from 12 m C57Bl, 6 m and 12 m BlAJ and 12 m+ONX BlAJ mice. Samples were resolved on polyacrylamide gels (ranging from 6 to 12%), transferred to nitrocellulose membranes (Bio-Rad Laboratories, California, USA) and overnight incubated with following antibodies: vinculin (1:600, MA5-11690, Invitrogen); PTX3 (C-10 1:600, sc-373951, Santa Cruz Biotechnology); p38 (1:500, E-AB-32460, Elabscience); PSMB5 (1:500; ab3330, Abcam); PSMB8 (1:500, Proteasome 20S LMP7, ab3329, Abcam); PSMB9 (1:500; Proteasome 20S LMP2 (EPR13785) ab184172, Abcam); S100β chain (C-3) (1:500, sc-393919, Santa Cruz Biotechnology); TNFα (1:500, E-AB-40015, Elabscience); HMGB1 (HAP46.5) (1:600, sc-56698, Santa Cruz); GAPDH (0411) (1:600, sc-47724, Santa Cruz); TLR4 (1:500, sc-293072, Santa Cruz); phosphoP38 (1:500, E-AB-20949, Elabscience); ERK1-2 (1:500, ab54230, Abcam); phosphoERK1-2 (1:500, E-AB-20868, Elabscience); AMPK-1 α (1:500, sc-74461, Santa Cruz); phosphoAMPK-α1/2 (1:600, STJ-90735, STJohnlabs); GSK-3 αβ (1ː500, sc-81496, Santa Cruz); TLR2 (1500, orb229137, Biorbyt): mTOR (1:500, PA5-34663, Invitrogen); PKCα (1:600, 610108, BD); CDK4 (1:500, sc-23896, Santa Cruz); Cyclin D1 (1:500, sc-8396, Santa Cruz); Cyclin E (1:500, sc-377100, Santa Cruz); MMP2 (1:500, ab37150, Abcam); MMP9 (1:500, ab38898, Abcam); adiponectin (1:500, 710179, Invitrogen); ATG-7 (1:500, sab4200304, Sigma-Aldrich); LC3B (1:500, L7543, Sigma-Aldrich); DRP1 (1:500, AB184247, Abcam); OXPHOS (1:500, MS604-300, Abcam); PGC1α (1:500, sc-518038, Santa Cruz); P62 (1:500, P0067, Sigma-Aldrich); RAGE (1:500, NBP2-03950, Novusbio); MYD88 (1:500, 23230-1-AP, Proteintech); AKT1/2/3 (1:500, ab126811, Abcam); Perilipin-1 (1:600, ab3526, Abcam); C3aR1 (1:100, PA5-109467 Invitrogen); pAkt (Ser473)(1:600, sc-81515, Santa Cruz Biotechnology); IKK-I (1:600, sc-376114, Santa Cruz Biotechnology); C5aR1/CD88 (1:600, 21316-1-AP, Proteintech); GPx1 (1:600, ab22604, Abcam). Filters were detected with peroxidase conjugated secondary antibodies (Agilent Technologies, California, USA) and developed by ECL (Amersham Biosciences, United Kingdom). Bands were quantitated in ImageJ software.

### In vitro ONX-0914 treatment of dysferlin-deficient Mø cells

12 m BlAJ mice (*n* = 5) were anesthetized with isoflurane and sacrificed by cervical dislocation. Femur bones (with hip and knee joints intact), dissected under semi-sterile conditions, were freed of any attached tissue, briefly dipped in 70% ethanol to dehydrate any remaining muscle tissue, then rinsed in D-PBS without Ca++/Mg++ (Gibco) before cutting off the two extremities. The bone marrow was extracted into ice-cold PBS by pushing it out of the bone cavity with a syringe equipped with a 25G needle, centrifuged at 800 rpm for 5 min and resuspended in macrophage complete medium. Bone marrow precursor cells were differentiated into Mø in RPMI-1640 medium (Thermo Fisher Scientific) supplemented with 20 ng/ml recombinant M-CSF (PeproTech), 2mM L-glutamine (Thermo Fisher Scientific), 10 mM HEPES (Thermo Fisher Scientific), 50 mm 2-Mercaptoethanol (Thermo Fisher Scientific), 10% FBS. Cells were routinely cultured at 37 °C in 21% O_2_ and 5% CO_2_ and regularly tested for the presence of mycoplasma. For immunofluorescence, cells were seeded in 12-well plates onto 18 mm diameter coverslips. For western blot analysis cells were collected after trypsinization and lysed in NP-40 lysis buffer to obtain whole protein lysate. For Mø polarization, recombinant murine TNFα (Sigma-Aldrich) was added at a concentration of 20 and 40 ng/ml for 16 h, followed by the addition of ONX-0914 at a final concentration of 300 nM to the same wells. Mø polarization was monitored using an inverted phase contrast microscope (Nikon TS100), and cells were generally cultured for ~48 h after the addition of TNFα.

### Histological and Immunofluorescence analysis of muscle sections

Histological and immunofluorescence analysis were performed on murine tissue sections. Muscular biopsies were collected from 6 m BlAJ, 12 m BlAJ and 12 m BlAJ+ONX, frozen in liquid-nitrogen cooled isopentane and cut on a cryostat into 10 µm slices. Human biopsies were frozen in liquid-nitrogen cooled isopentane and cut on a cryostat into 7 µm slices. H&E staining were performed as in [98] to evaluate the morphology of muscles. Oil Red O (ORO) staining was performed to determine the amount of lipids. For immunofluorescence staining, sections were fixed with 4% paraformaldehyde for 10 min, permeabilized with 0.1% Triton X-100 for 10 min and incubated with 10% donkey serum to block non-specific binding for 1 h. Slides were then incubated with the primary antibodies (overnight at 4 °C) diluted in blocking solution at the following dilutions: PTX3 (MNB1) (1:50, ab90806, Abcam); CD31 (1:50, ab119339, Abcam); Mannose Receptor (CD206) (1:50, ab64693, Abcam); Iba1/AIF-1 (E404W) antibody (1:50, Cell Signaling Technology); ER-TR7 (1,50, NB 100-64932, Novus); Isolectin GS-IB4 Alexa Fluor™ 594 Conjugate (1:100, I21413, Thermo Fisher Scientific); WGA (Wheat Germ Agglutinin) Alexa Fluor 488™ Conjugated (1:50, W11261, Thermo Fisher Scientific); α-Smooth Muscle – FITC (1:100, F3777, Sigma-Aldrich); neuron-glial antigen 2 (NG2) (1:50, AB5320, Millipore); C3aR1 and C5aR1/CD88 (1:50, 21316-1-AP, Proteintech). PDGFRβ (1:50, 28E1, Cell Signaling). Fluorochrome-conjugated secondary antibodies were diluted 1:200 in PBS1X and added for 1 h at room temperature. Slides were then mounted with Prolong Gold® Antifade Reagent with DAPI (Thermo Fisher, Carlsbad, CA). Images were acquired by an inverted fluorescence microscope Dmi800, while Z-stack and tile reconstructions were acquired by confocal microscope Sp8 (Leica). For H&E, ORO, and ARS, images were captured by Leica microdissector (CTR6000). Quantification of fluorescence was performed by means of ImageJ Software (NIH). Threshold color Plug in of ImageJ Software was used to quantify the amount of tissue stained with Alizarin Red and Oil red. Data were analyzed by GraphPad Prism^TM^ and expressed as means ± SD. ImageJ software was used to estimate the percentage of Mac1 and Mac2 in *n* = 12 images from each mouse, with *n* = 5 mice/group. For cells and vessels expressing CD31, isolectin and α-SMA, quantification of immunoreactivity was performed in *n* = 11–15 images from each mouse, with *n* = 5 mice/group.

### Serum analysis

CPK analysis was performed on serum samples of 12 m BlAJ, 12 m BlAJ+ONX and 12 m C57Bl mice with CPK kit (Cobas), according to manufacturer’s instructions. C3 and C5 serum levels of the same mice were measured using a commercially available mouse C3 ELISA Kit (catalog no. MBS135930; MyBioSource) and C5 ELISA kit (catalog no. ab264609; Abcam) according to the manufacturer’s instructions.

### Muscle functional measures

Treadmill test was performed as follow: after 10 and 20 min of training at constant velocity, the next day 12 m BlAJ and 12 m BlAJ+ONX mice run for 20 min with increasing velocity stating from 20 m/s. Tetanic force of TA of treated and untreated 12 m BlAJ mice was determined as described in [41] and expressed in kN/m^2^.

### Qualitative (RT-qPCR) experiments

Total RNA was extracted from skeletal muscles of C57Bl and AJ mice and cDNA generated using the Reverse Transcriptase Kit (Thermo Fisher Scientific, California, USA). All the samples were tested in triplicate and the threshold cycles (Ct) of target genes were normalized against the housekeeping gene, glyceraldehyde 3-phosphate dehydrogenase (GAPDH). The expression of genes was quantified by means SYBR-Green method. Relative transcript levels were calculated starting from the Ct values as X = 2^−ΔΔct^ where X is the fold difference in amount of target gene versus GAPDH and ΔΔCt = ΔCt_target_ − ΔCt_GAPDH_. The sequence of primers used is listed in Table 3. The expression of MRF-4 (Mm00435126) was calculated with the Probe Mix by Applied Biosystem.

**Table 3:** List of RT-qPCR primers.

Primer	Sequence 3’-->5’
mMurF1-r	TGAGGCAGAGTCTCTCTATGT
mRORγt-f	GACTGACAATCAGCAGGGATAA
mRORγt-r	GGGAAATACAATGAGGTATTGAAAGG
mp62-f	AGGCGCACTACCGCGAT
mp62-r	CGTCACTGGAAAAGGCAACC
mPPARα-f	TGATTGGTTCCAGGCAATTAGA
mPPARα-r	CACTCGTACAGTCAGTTCAGTC
mpdk4-f	GTCTCAATAGTGTCACCTGTGTAA
mpdk4-r	CCTGGGCATTTAGCATCTATCT
mPdh-f	GAAGGCCCTGCATTCAACTTC
mPdh-r	ATAGGGACATCAGCACCAGTGA
mGlut4-f	CCTGCTTGGCTTCTTCATCT
mGlut4-r	GGTTTCACCTCCTGCTCTAAA
mPfk-f	CAGTCAGTGCCAACATAACCAA
mPfk-r	CGGGATGCAGAGCTCATCA
MCAD-f	CGGCTTGTCAAGGAAGAACT
MCAD-r	CAGCACAGAAATGCTGCTATG
SERCA2α-f	TAGGCCTCCAGTCCTAACTT
SERCA2α-r	CCAACATCTGTCTACTGCTTCT

### Statistics

To determine significance when comparing multiple groups’ means, we used one-way ANOVA followed by Tukey’s multiple comparison test; Student’s *t*-test was used to compare two groups assuming equal variances. In case of non-parametric test, we performed the Kruskal–Wallis test. For repeated measures, statistical significance was calculated via simple linear regression, by testing for differences between slopes of best-fit lines. A 95% confidence band of each best-fit line is shown. In any cases, the difference among groups was considered significant as follow: * at *p* < 0.05; ** at *p* < 0.01; *** at *p* < 0.001; **** at *p* < 0.0001. Sample size was determined by using a sample-size calculator freely available on internet. All the samples that did not rich quality control standards due to the presence of contaminants for RNA or to problems in freezing procedures for histological analysis were excluded.

## Supplementary information

Supplementary Table 1

AJ checklist

Supplementary Figures and Legends

Supplementary Figure 1

Supplementary Figure 2

Supplementary Figure 3

Supplementary Figure 4

Supplementary Figure 5

Original Data File

Original Data File

## Data Availability

The data used to support the findings of this study are available from the corresponding author upon reasonable request.
