# Supplementary material for: Inhibition of the immunoproteasome modulates innate immunity to ameliorate muscle pathology of dysferlin-deficient BlAJ mice
Source: Cell Death Dis. 2022 Nov 19;13(11):975. doi: 10.1038/s41419-022-05416-1 (PMC9675822; doi:10.1038/s41419-022-05416-1)
Supplement: Supplementary file 3 — Supplementary Figures and Legends [file 41419_2022_5416_MOESM3_ESM.docx]

**Supplementary Figure 1. Evaluating the amount of inflammatory and immune cells in skeletal muscle and spleen of 12m C57Bl and 12m BlAJ mice.**


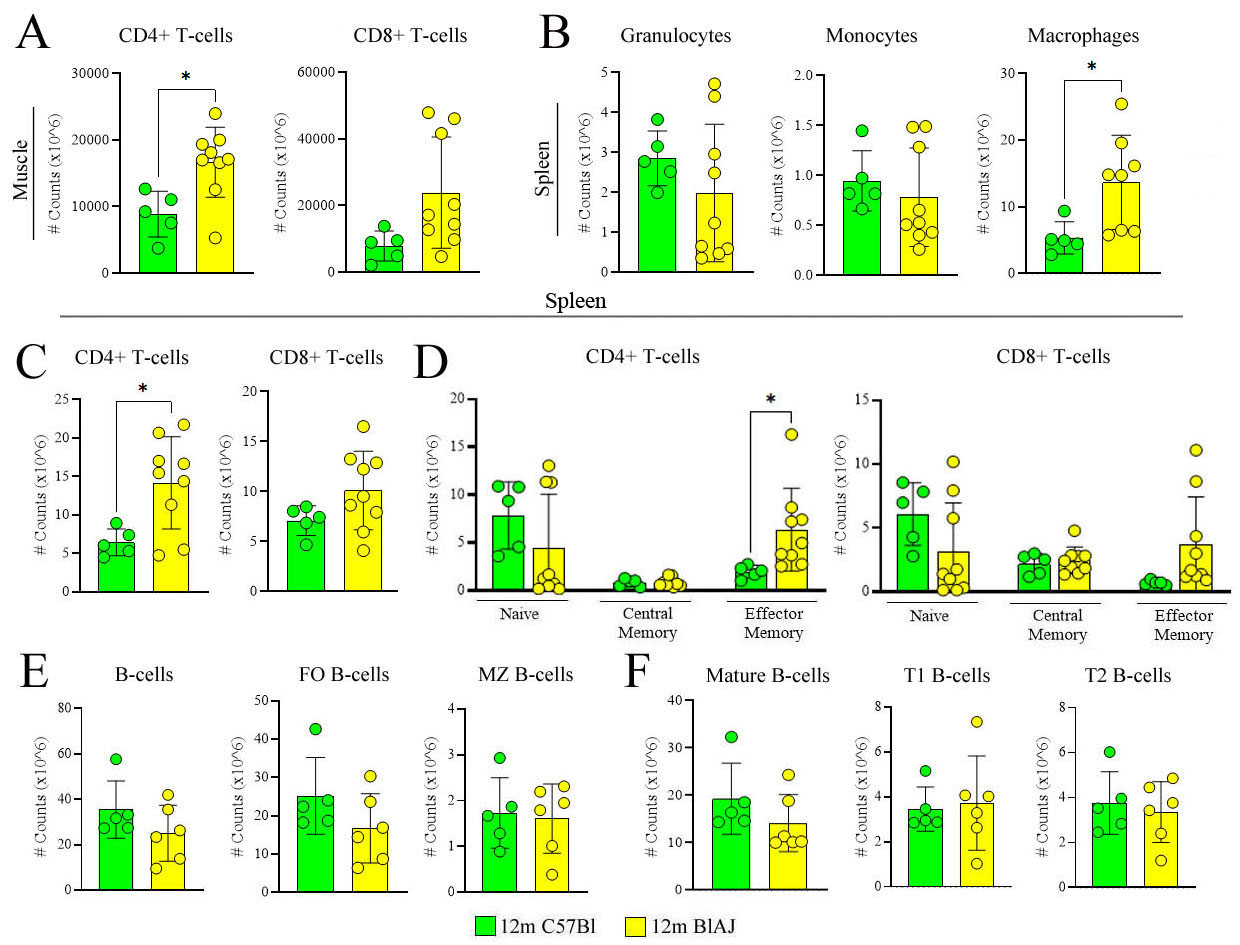


Representative FACS analysis of the amount (expressed in millions of cells) of muscle CD4+/CD8+ T-cells (A); splenic macrophages, monocytes and granulocytes (B); CD4+/CD8+ T-cells (C) and CD4+/CD8+ T-cell subsets (D); total, FO and MZ B-cells (E); mature, transitional T1 and T2 B-cells (F) of 12m C57Bl and 12m BlAJ mice.

Data are presented as mean±SD of n=3 independent experiments with n=3-12 animals/group. Student t-test: *p<0.05.

**Supplementary Figure 2. Evaluating the amount of inflammatory and immune cells in skeletal muscle and spleen of 12m BlAJ and 12m BlAJ+ONX mice.**


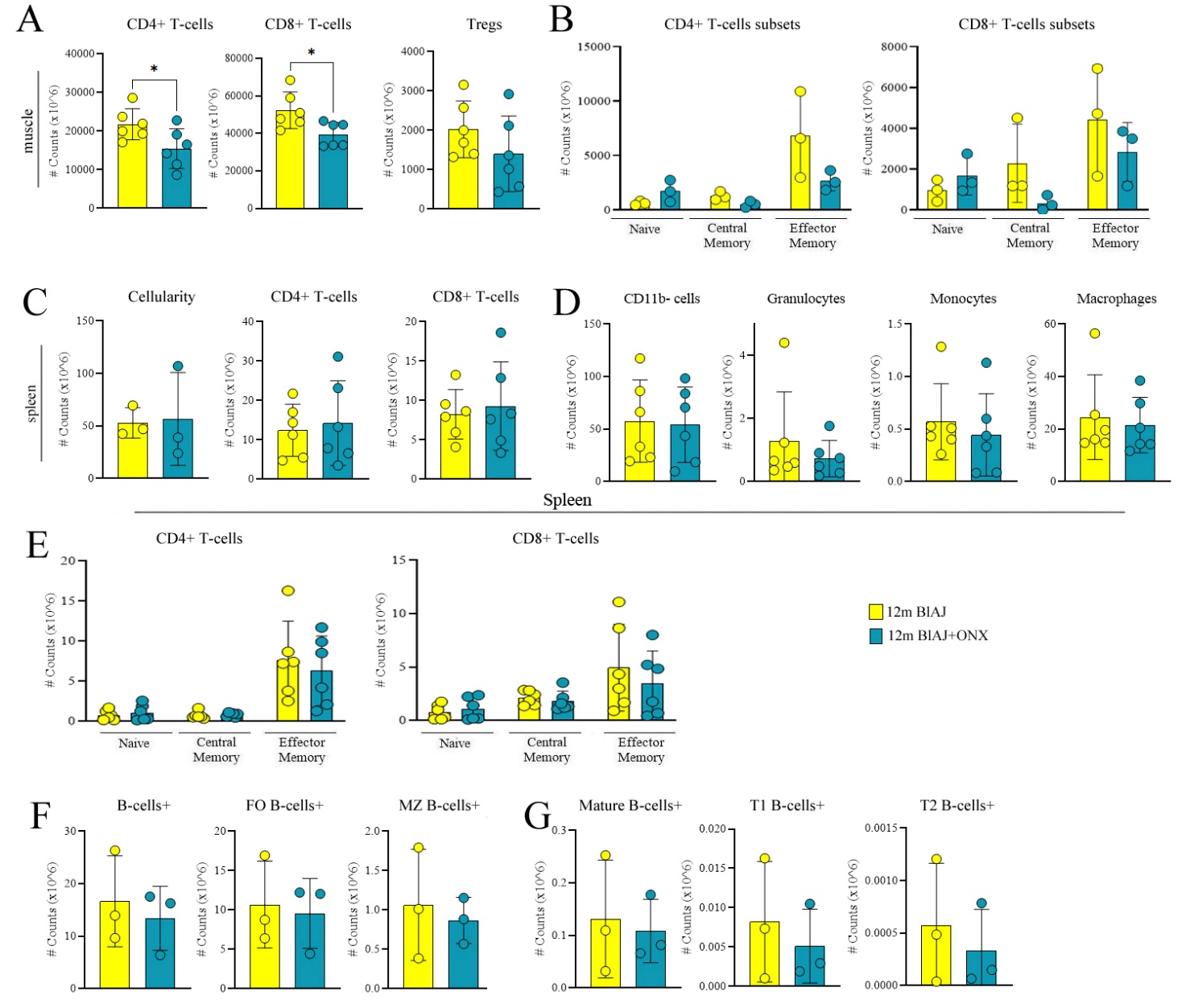


Representative FACS analysis of the amount (expressed in millions of cells) of muscle CD4+/CD8+ T-cells and Tregs (A) and CD4+/CD8+ T-cell subsets (B). Spleen cellularity and total amount of CD4+/CD8+ T-cells (C); CD11- cells, macrophages, monocytes and granulocytes (D); CD4+/CD8+ T-cell subsets (E); total, FO and MZ B-cells (F); mature, transitional T1 and T2 B-cells (G) of 12m BlAJ and 12m BlAJ+ONX mice.

Data are presented as mean±SD of n=3 independent experiments with n=6 animals/group. Student t-test: *p<0.05.

**Supplementary Figure 3. Evaluating the inflammatory features of 12m BlAJ mice.**


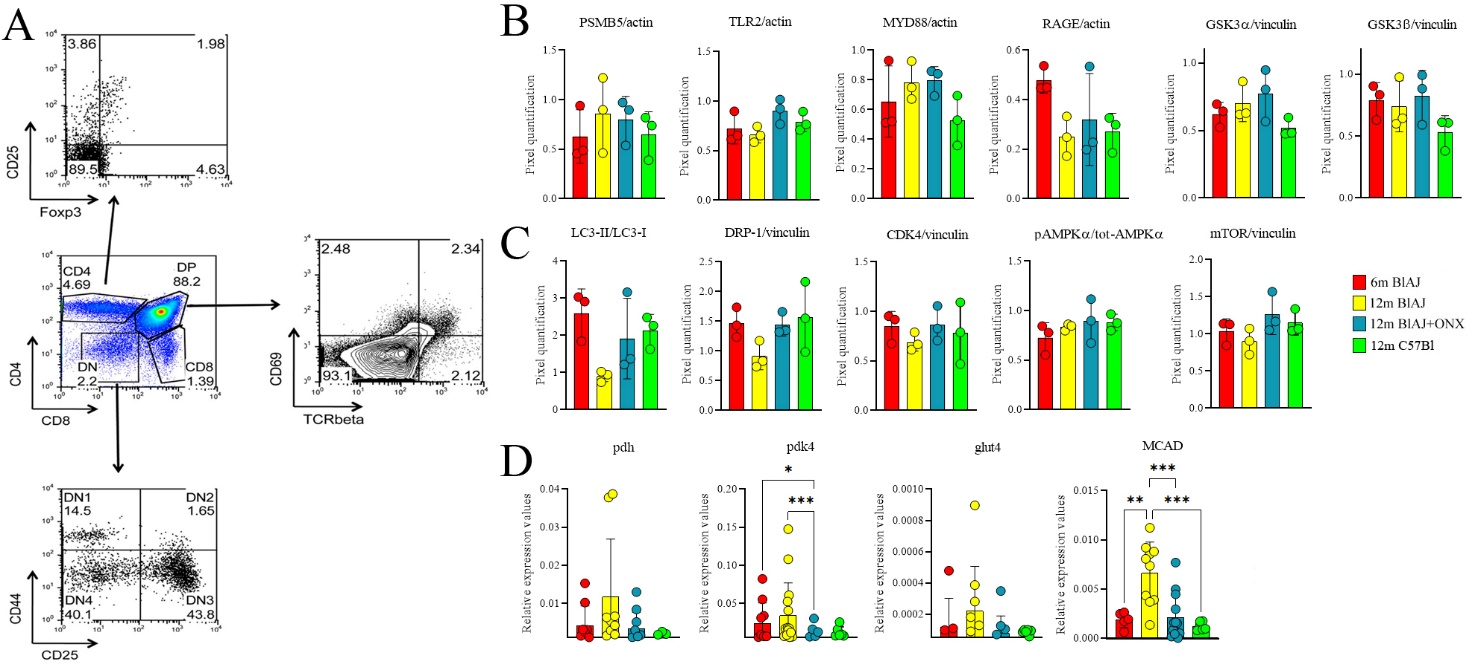


Representative FACS profile is shown. The numbers within the panels indicate the percentage of each population of live cells from thymus according to the expression of CD4 and CD8. Within the CD4-/CD8- gate, four populations are separated according to the expression level of CD44 and CD25. Within the CD4+/CD8+ gate, further populations are separated according to the expression level of CD69 and TCR-β while on CD4+ gate, further populations are separated according to the expression of CD25 and FoxP3 (A). WB analysis of inflammatory mediators in psoas of 6m BlAJ, 12m BlAJ and 12m BlAJ+ONX mice (B,C). RT-qPCR analysis of the expression levels of *pdk4, mcad, Glut4, pdh and pfk* in psoas of 6m BlAJ, 12m BlAJ and 12m BlAJ+ONX mice (D). Data are presented as mean±SD of n=3 independent experiments with n=3-9 animals/group (and technical replicates for RT-qPCR). One-way ANOVA: **p<0.01 and ***p<0.001.

**Supplementary Figure 4. Evaluation of proteomic expression in TNF-α/ONX-0914 treated monocytes isolated from 12m BlAJ femur.**


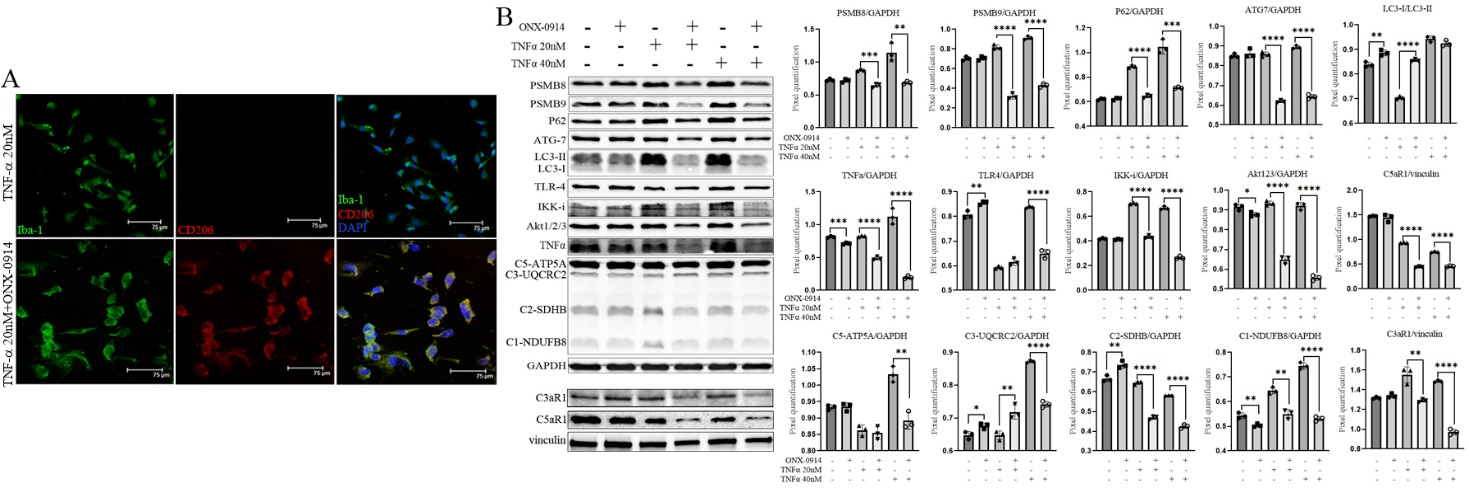


(A) Typical morphologies of “fried egg shape” M1 Mø was observed in the presence of TNFα. Immunofluorescence staining showed the co-expression of Iba1 (green) and CD206 (red) only in the ONX-0914 treated dysferlin-deficient Mø. (B) Proteomic analysis of macrophages treated *in vitro* with 20nM and 40nM TNF-α supplemented with ONX-0914 for the expression of immunoproteasome subunits; inflammatory mediators; complement cascade proteins. Data are presented as mean±SD of n=3 independent experiments. One-way ANOVA: *p<0.05, **p<0.01, ***p<0.001 and ****p<0.0001.

**Supplementary Figure 5. Gene expression profiles related to macrophage and T-cell function in 6m BlAJ, 12m BlAJ and 12m BlAJ+ONX-0914 mice.**


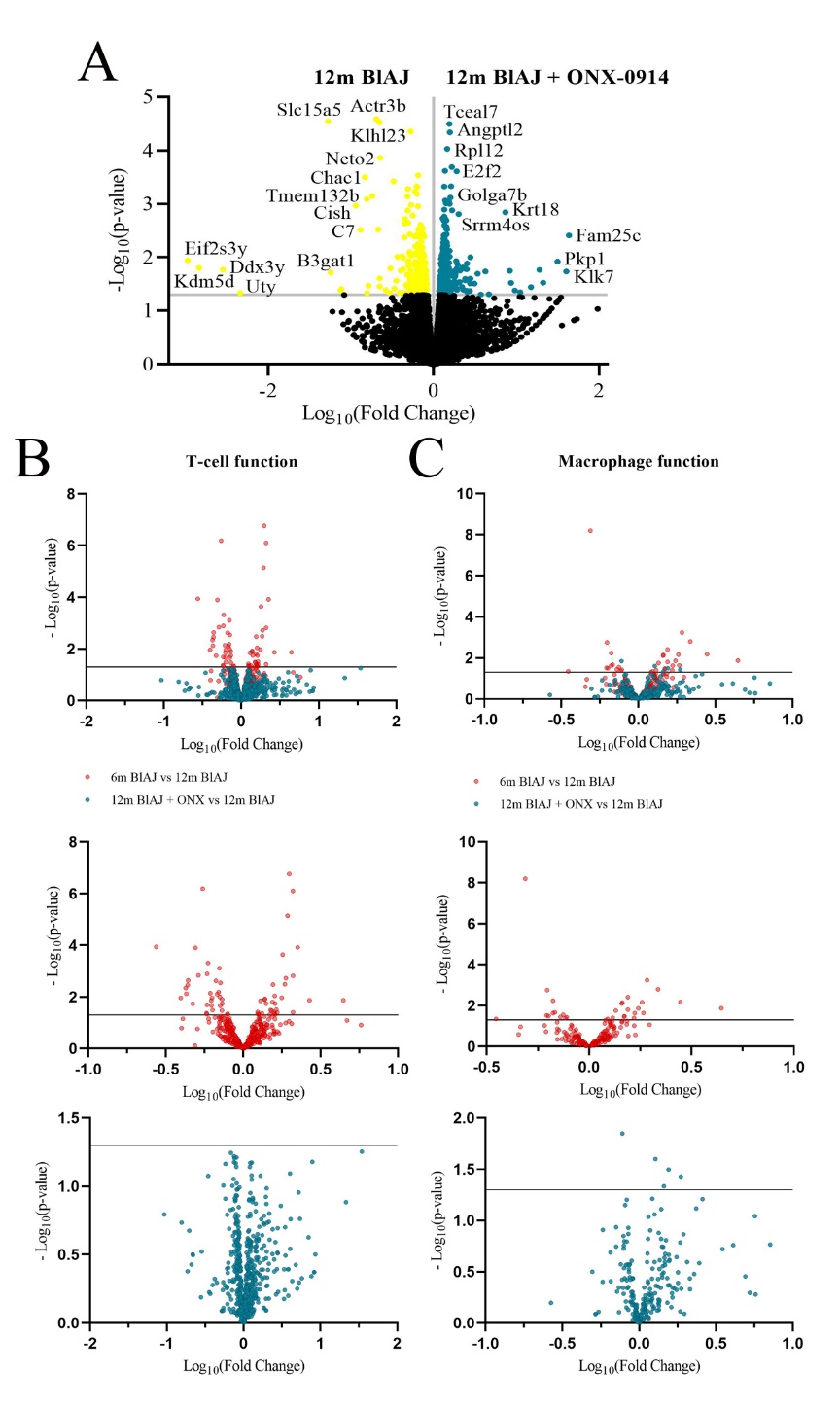


(A) Volcano plot analysis of genes differentially expressed in 12m BlAJ+ONX-0914 vs 12m BlAJ muscles: blue and yellow dots represent genes upregulated in 12m BlAJ+ONX-0914 and 12m BlAJ, respectively, with a p-value < 0.05 (corresponding to a -Log_10_(p-value) > 1.30). (B) Volcano plot analysis of genes involved in macrophage proliferation (Biological Process; GO: 0061517), differentiation (Biological Process; GO: 0030225), migration (Biological Process; GO: 1905517), activation (Biological Process; GO:0042116), and cytokine production (Biological Process; GO: 0010934). 6m BlAJ vs 12m BlAJ data series is represented via red dots. 12m BlAJ+ONX-0914 vs 12m BlAJ data series is represented via blue dots. Dots above the horizontal line at y=1.30 represent differentially expressed gene with a p-value<0.05 (corresponding to a -Log_10_(p-value)>1.30). Data series are plotted together (upper panel) and isolatedly (lower panels). (C) Volcano plot analysis of genes involved in T-cell proliferation (Biological Process; GO: 0042098), differentiation (Biological Process; GO: 0030217), migration (Biological Process; GO: 0072678), activation (Biological Process; GO: 0042110), cytokine production (Biological Process; GO: 0002369), and T-cell mediated immunity (Biological Process; GO: 0002369). 6m BlAJ vs 12m BlAJ data series is represented via red dots. 12m BlAJ+ONX-0914 vs 12m BlAJ data series is represented via blue dots. Dots above the horizontal line at y=1.30 represent differentially expressed gene with a p-value<0.05 (corresponding to a -Log_10_(p-value)>1.30). Data series are plotted together (upper panel) and isolated (lower panels).
