# Supplementary figures and images for: Inhibition of the immunoproteasome modulates innate immunity to ameliorate muscle pathology of dysferlin-deficient BlAJ mice
Source: Cell Death Dis. 2022 Nov 19;13(11):975. doi: 10.1038/s41419-022-05416-1 (PMC9675822; doi:10.1038/s41419-022-05416-1)

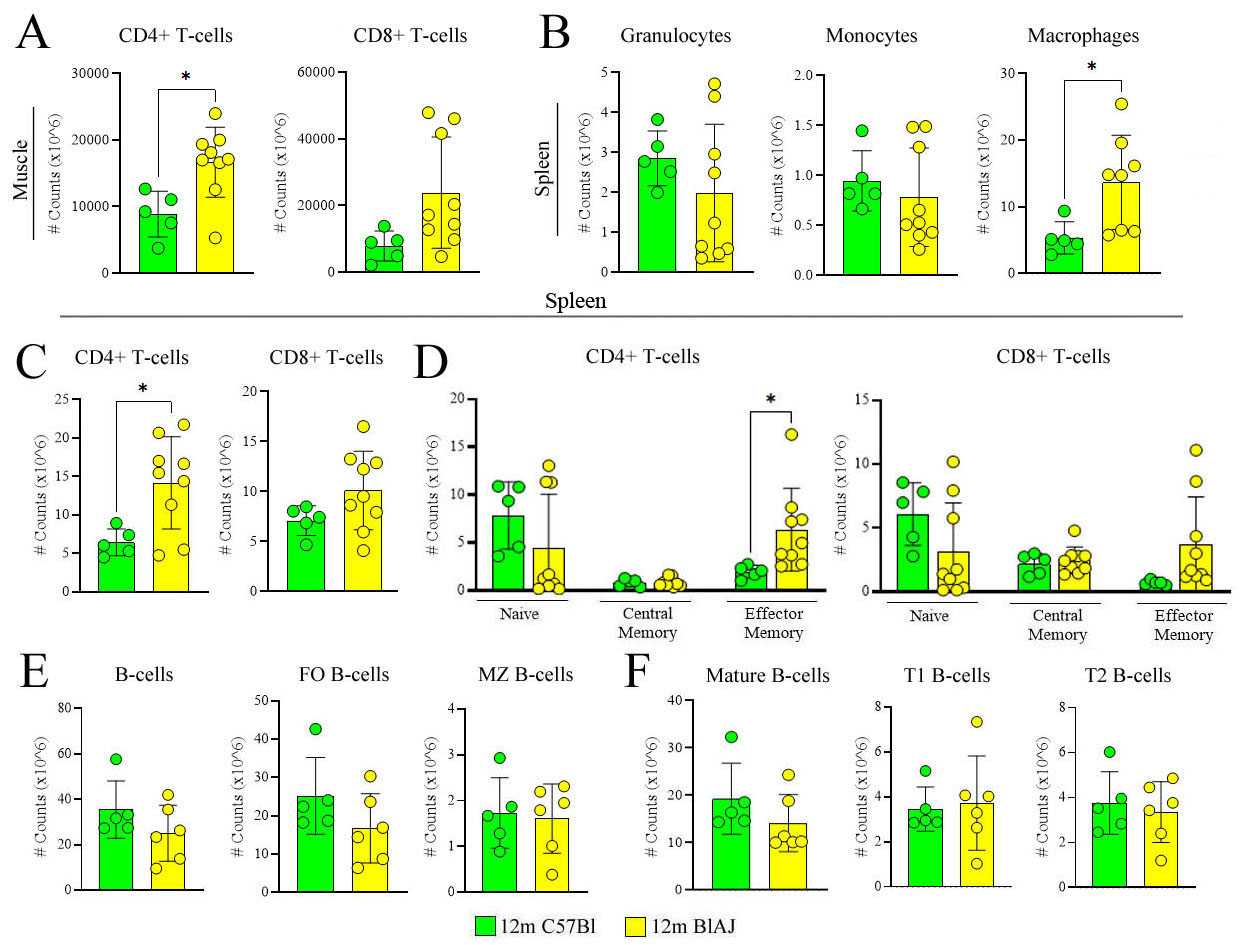

Supplement: Supplementary file 4 — Supplementary Figure 1 [file 41419_2022_5416_MOESM4_ESM.jpg]

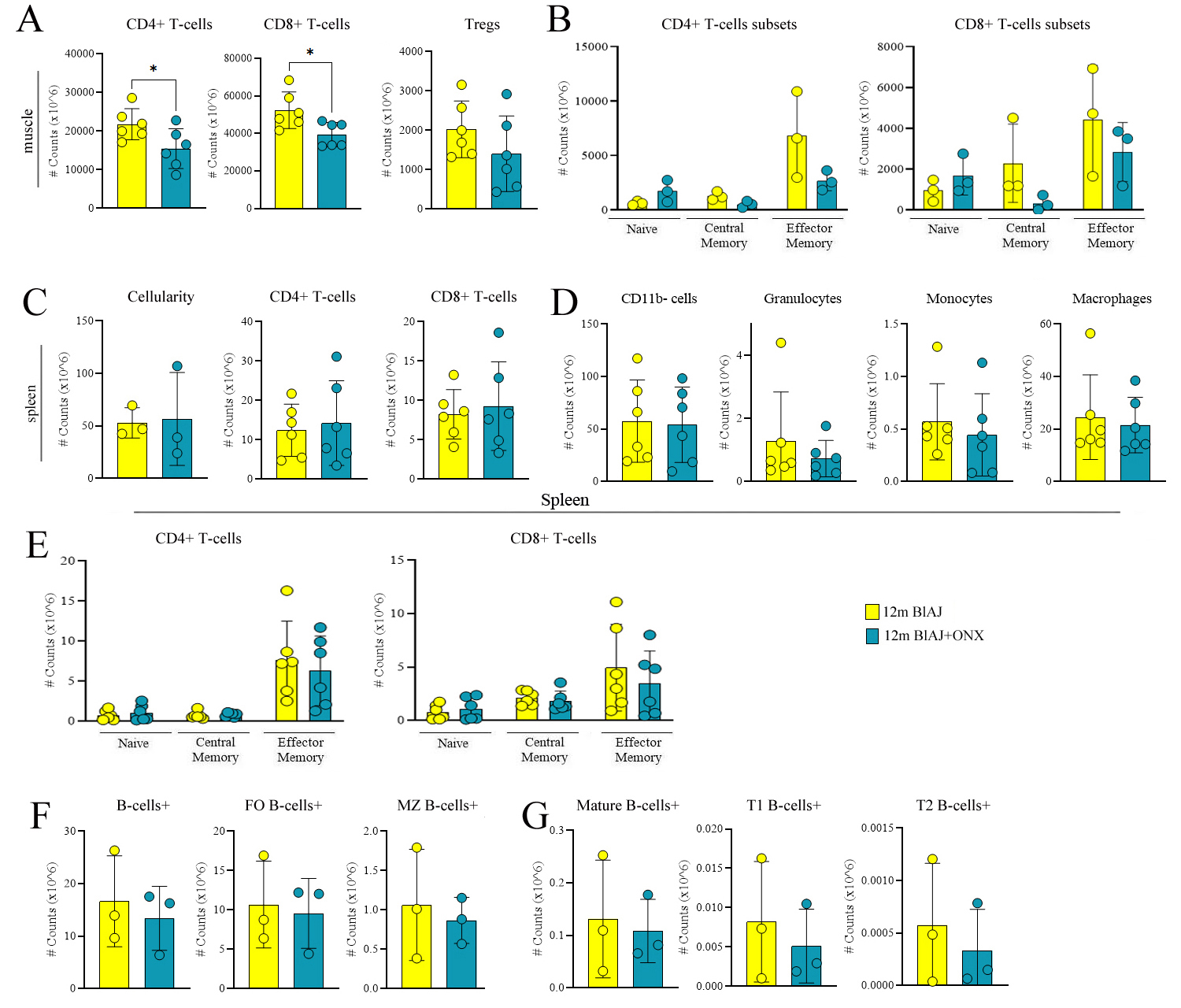

Supplement: Supplementary file 5 — Supplementary Figure 2 [file 41419_2022_5416_MOESM5_ESM.jpg]

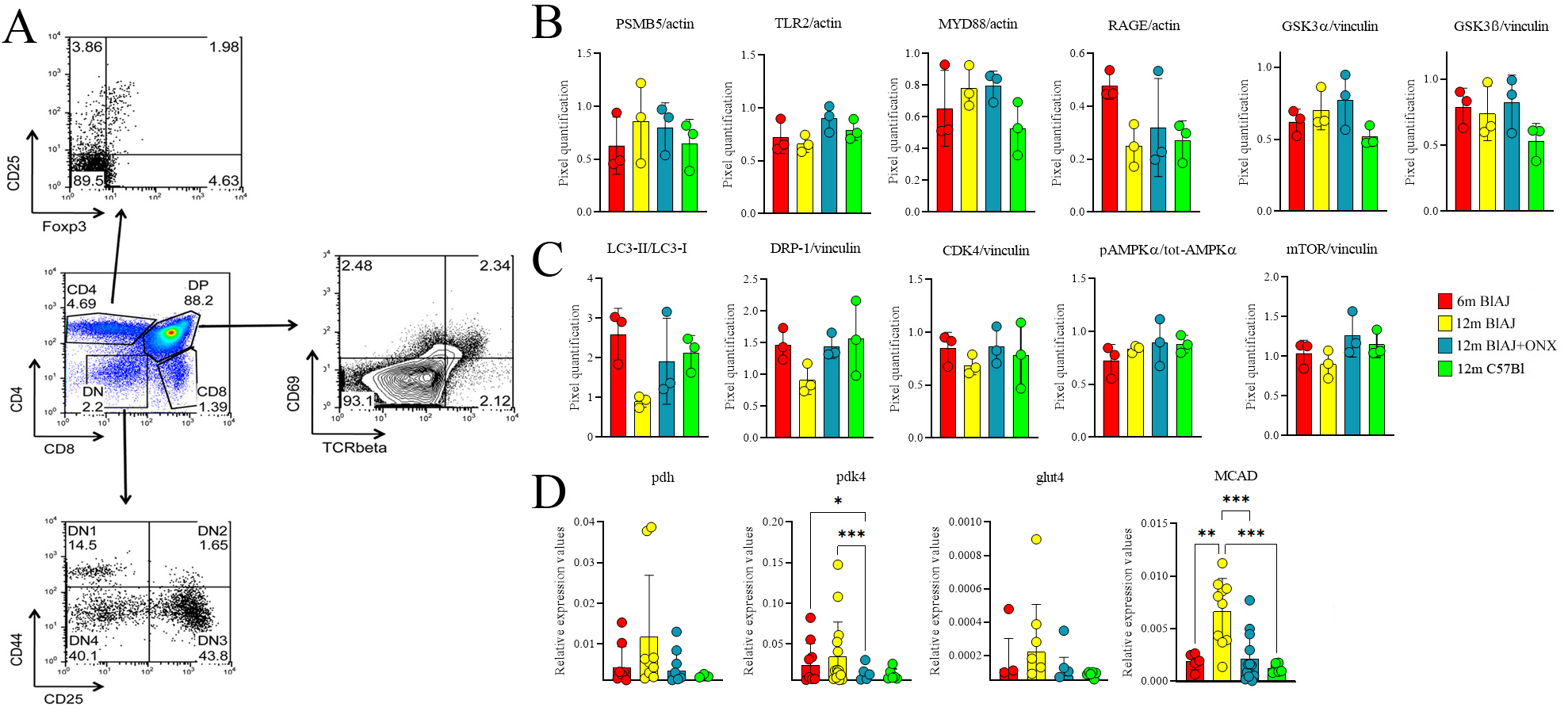

Supplement: Supplementary file 6 — Supplementary Figure 3 [file 41419_2022_5416_MOESM6_ESM.jpg]

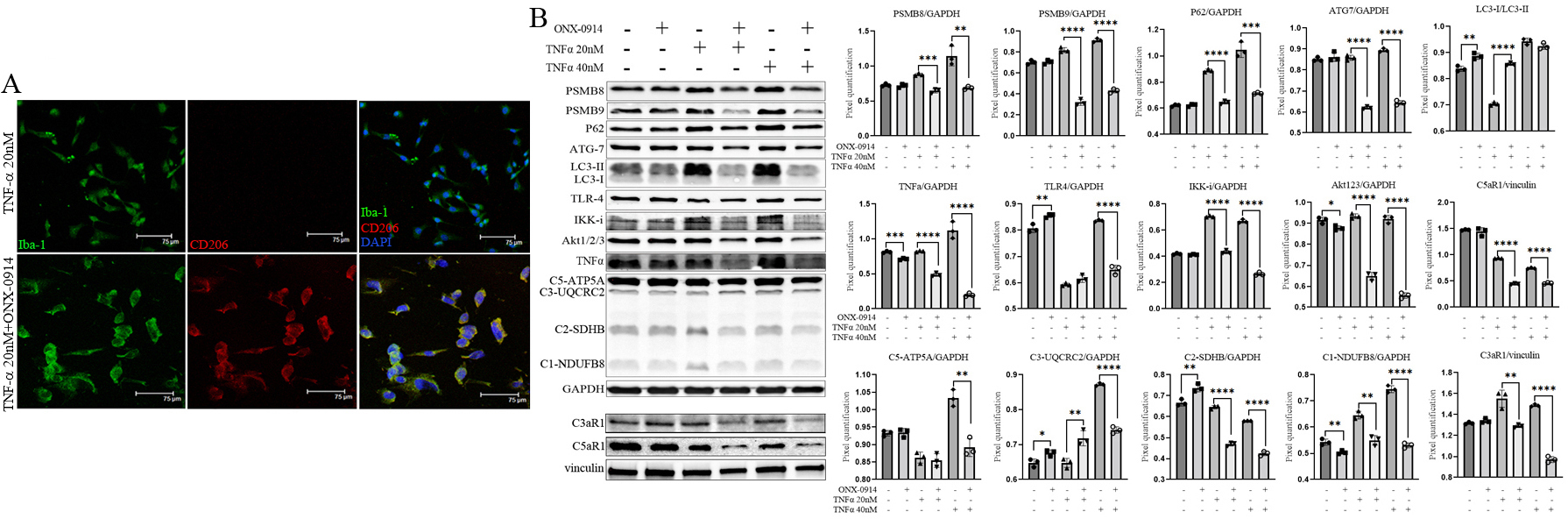

Supplement: Supplementary file 7 — Supplementary Figure 4 [file 41419_2022_5416_MOESM7_ESM.jpg]

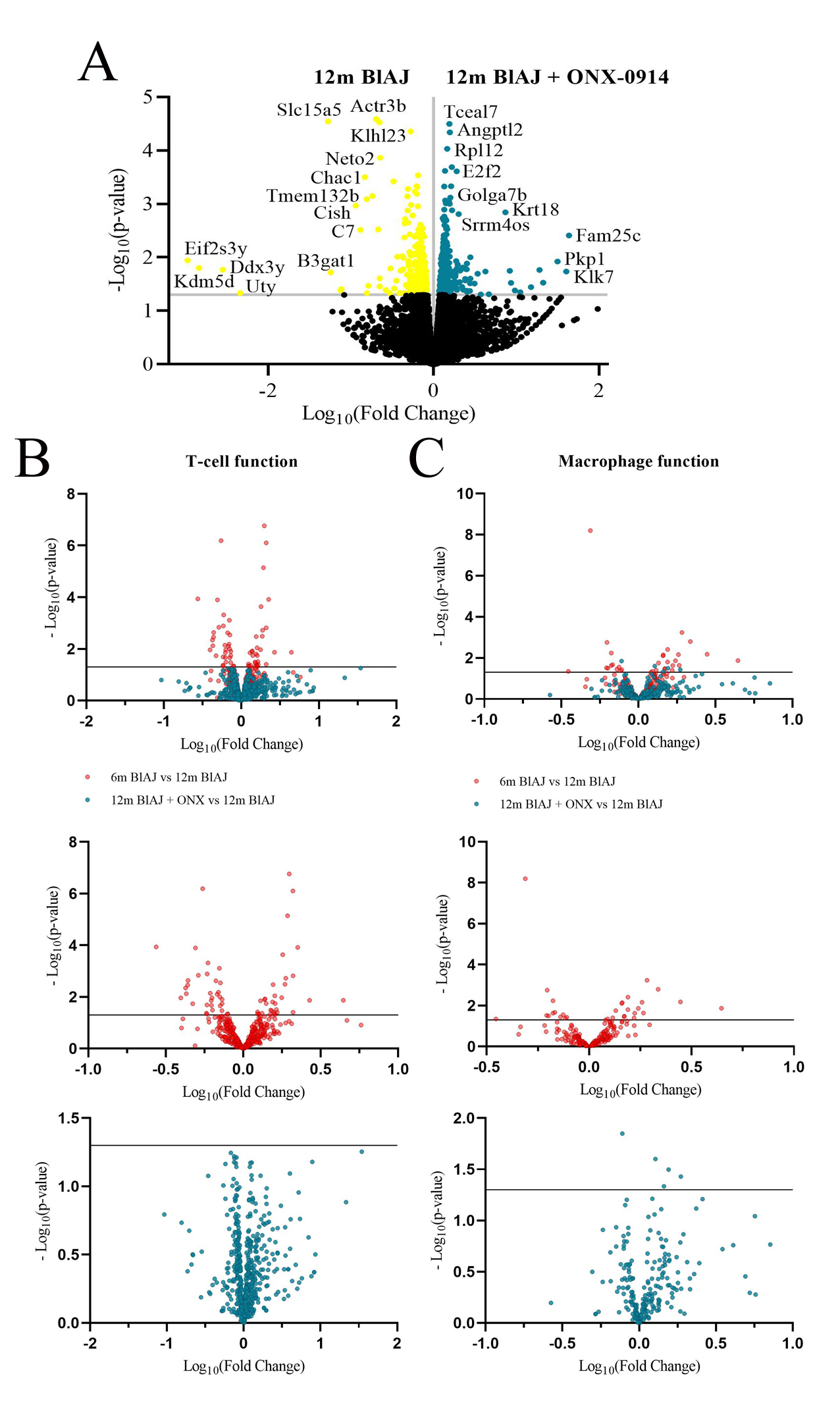

Supplement: Supplementary file 8 — Supplementary Figure 5 [file 41419_2022_5416_MOESM8_ESM.jpg]
