# Supplementary material for: Inhibition of the immunoproteasome modulates innate immunity to ameliorate muscle pathology of dysferlin-deficient BlAJ mice
Source: Cell Death Dis. 2022 Nov 19;13(11):975. doi: 10.1038/s41419-022-05416-1 (PMC9675822; doi:10.1038/s41419-022-05416-1)

Figure 1E

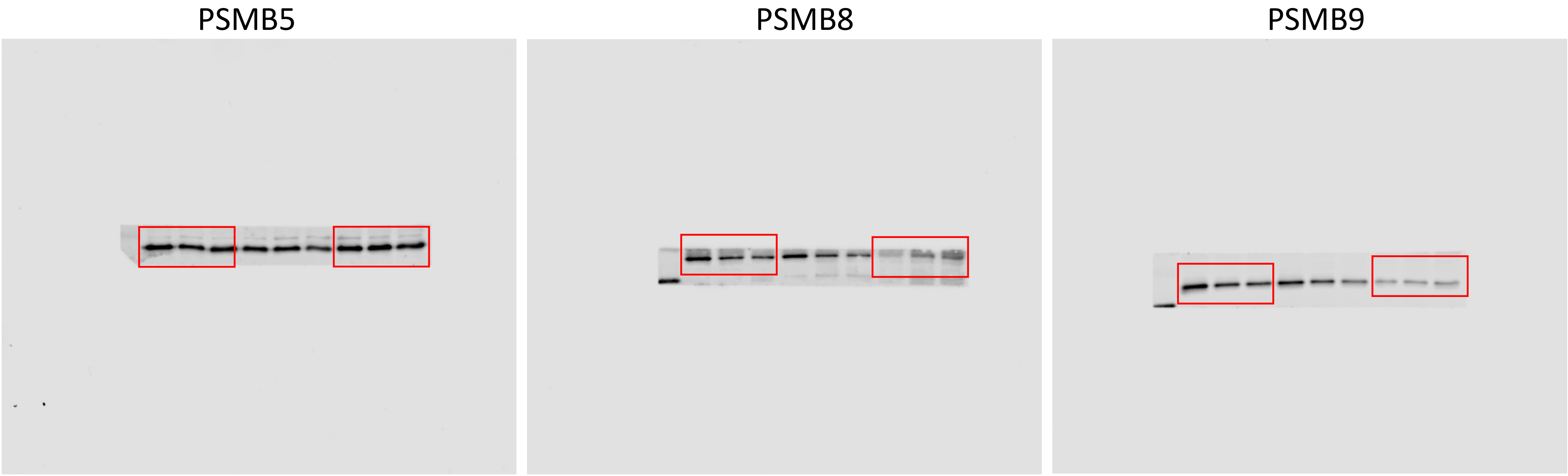

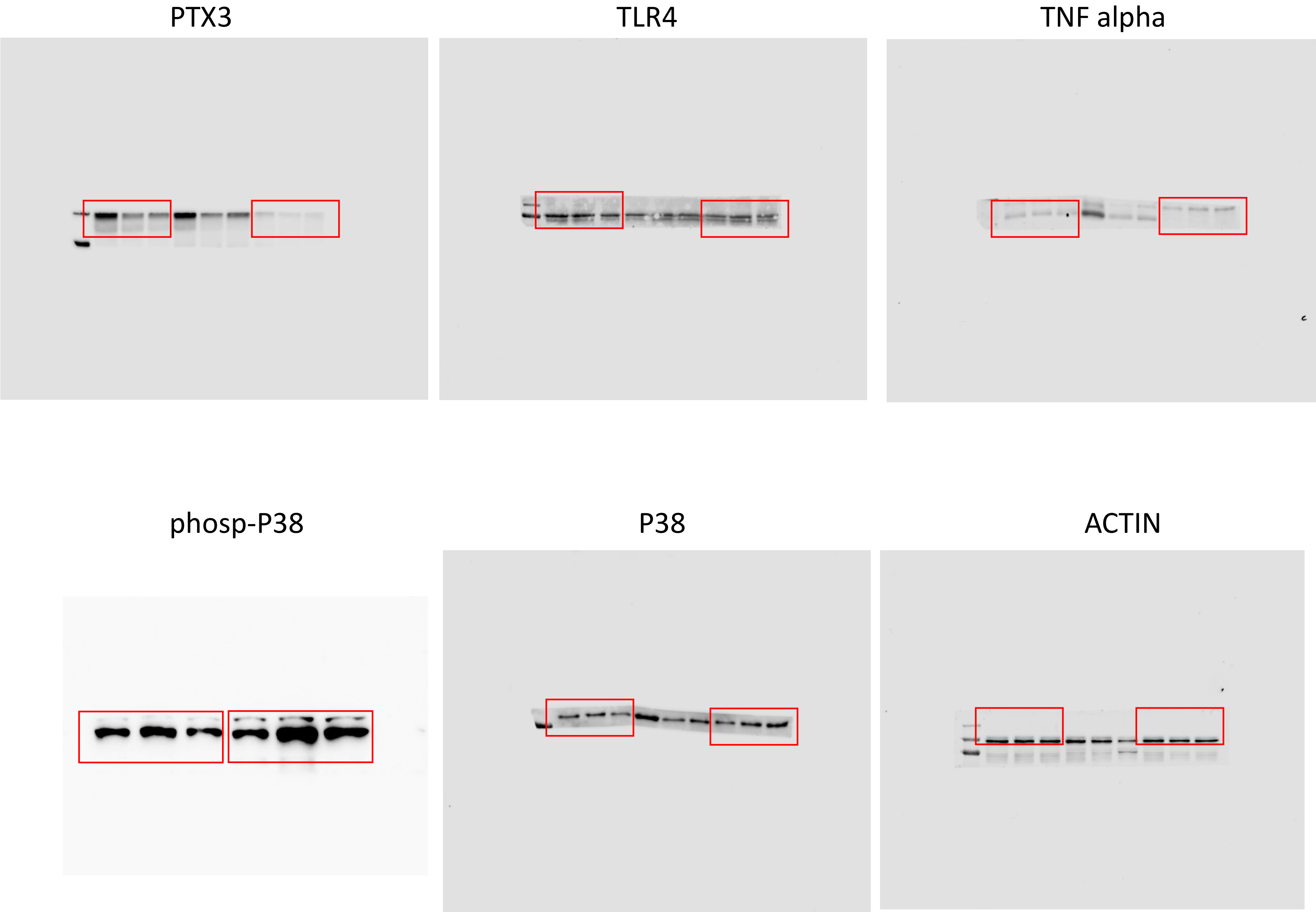
PTX3
TLR4
TNF alpha
phosp-P38
P38
ACTIN

phosp- AKT 1/2/3

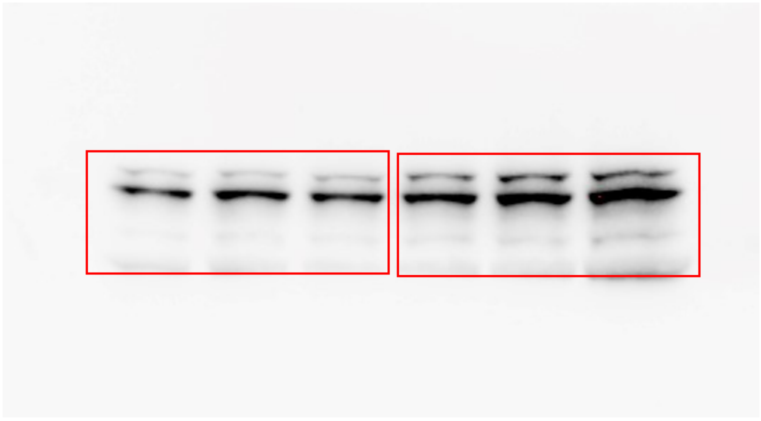

AKT 1/2/3

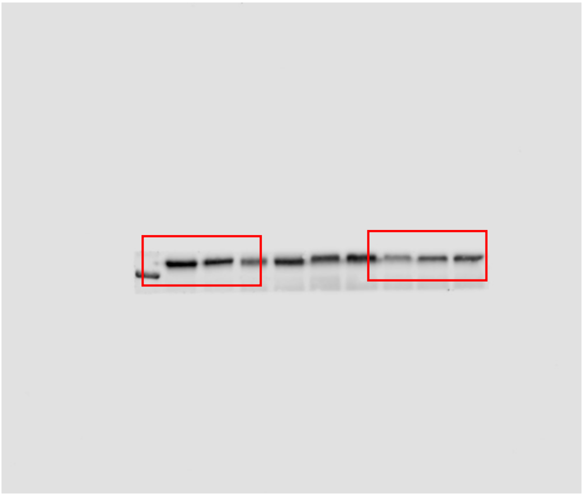

phosp- AMPKα

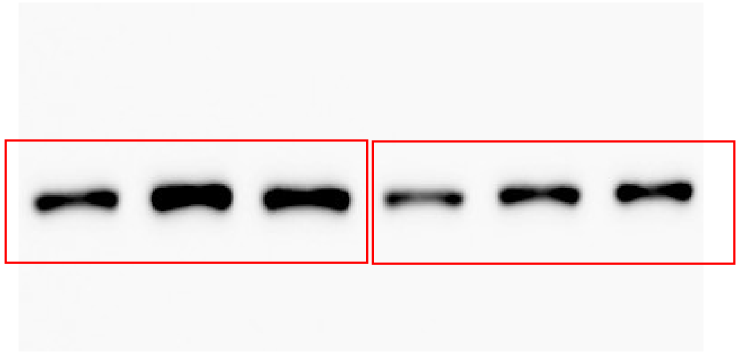

Figure 3A

PSMB8

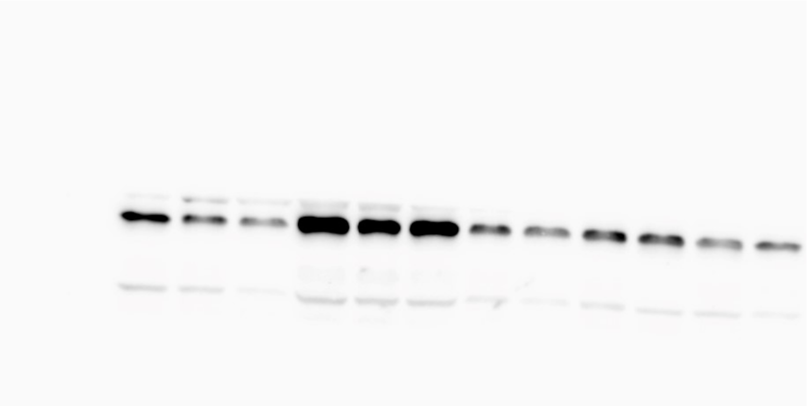

PSMB9

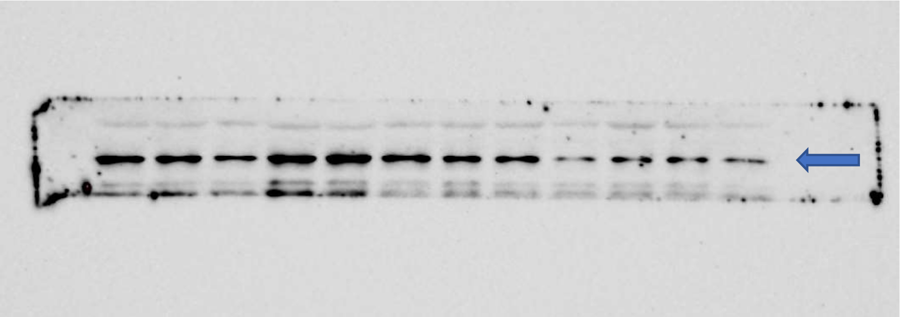

PSMB5

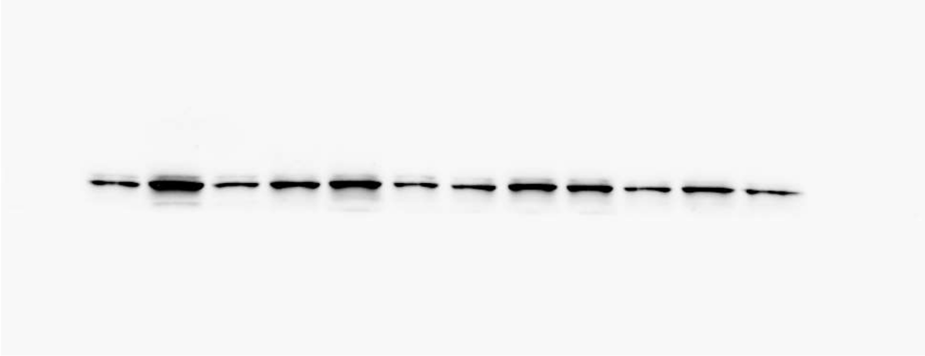

ACTIN

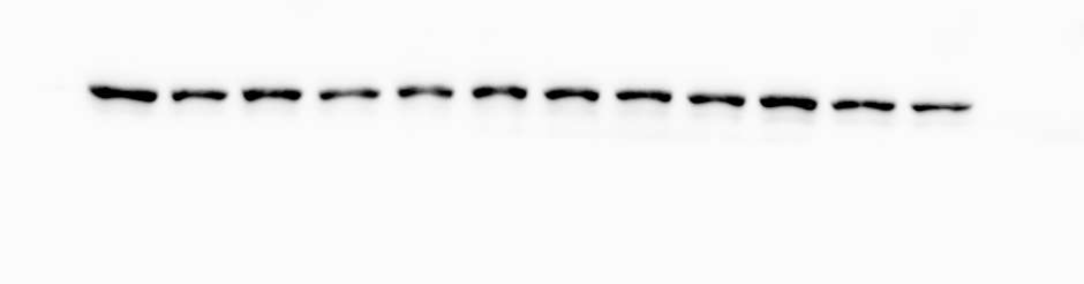

Figure 3B

MYD88

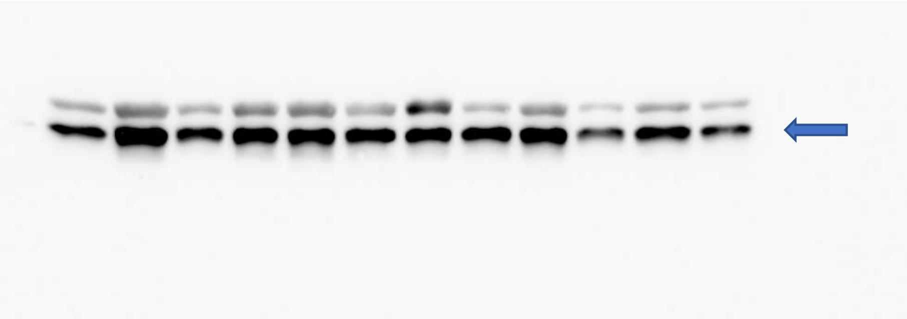

PTX3

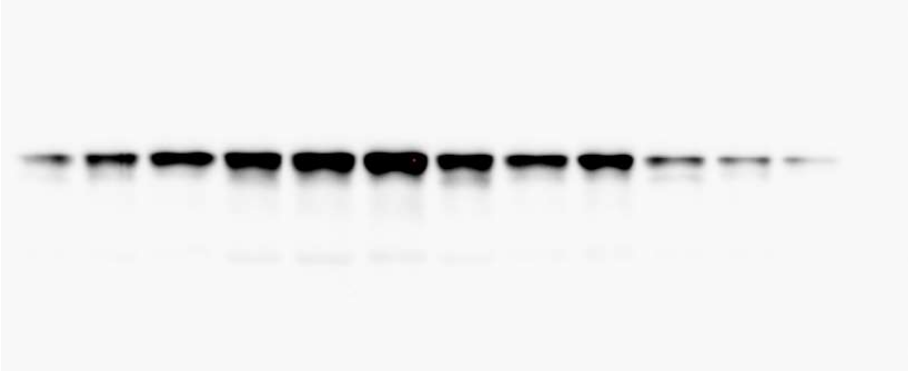

RAGE

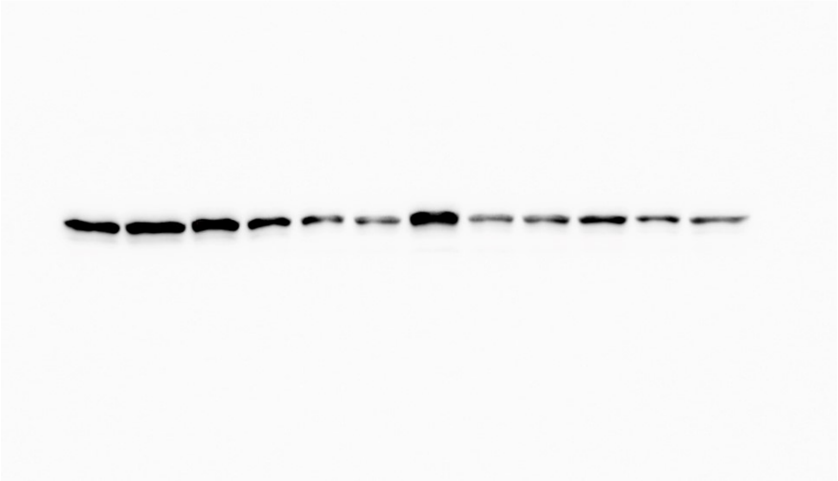

TLR2

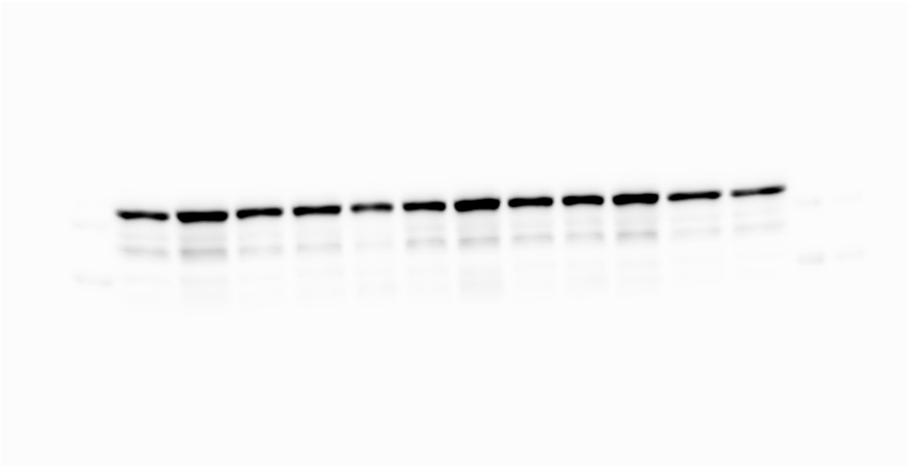

TLR4

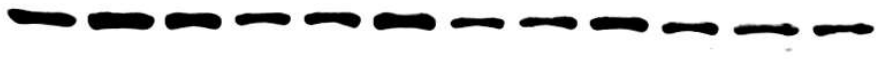

S100-β

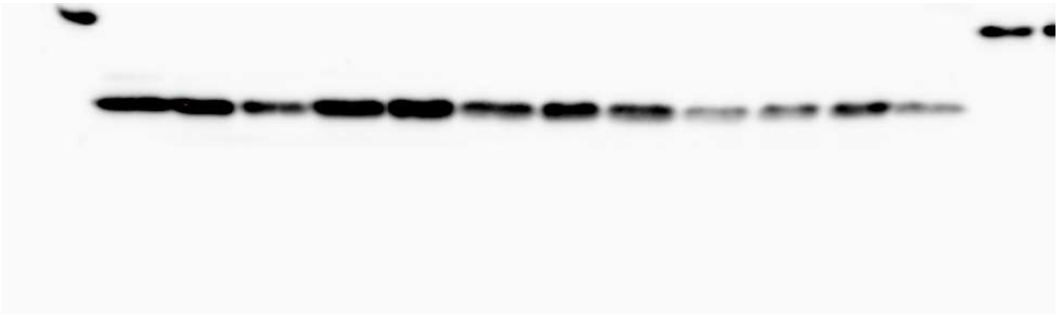

HMGB-1

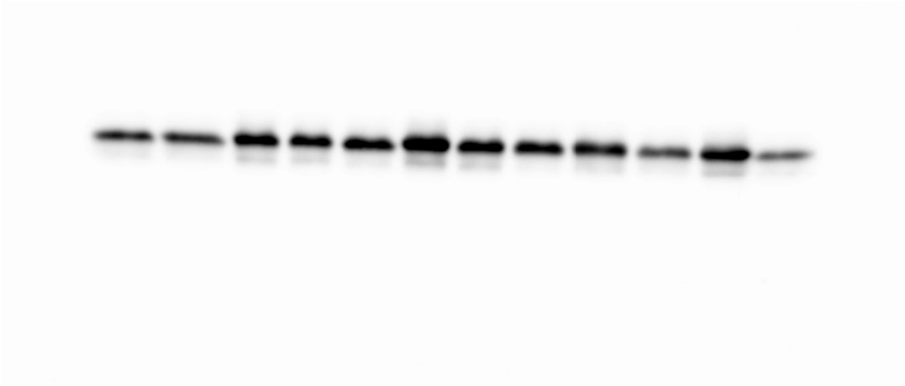

GP-x1

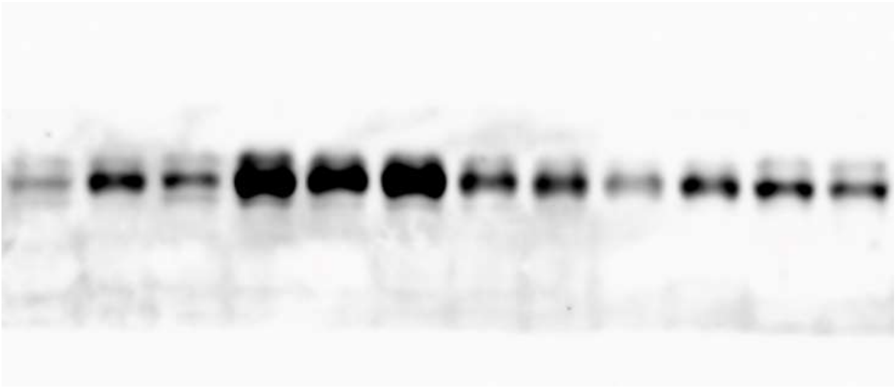

actin

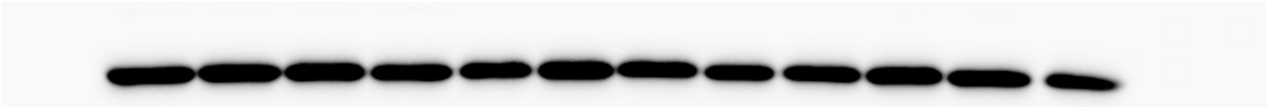

Figure 3C

Phospho-P38

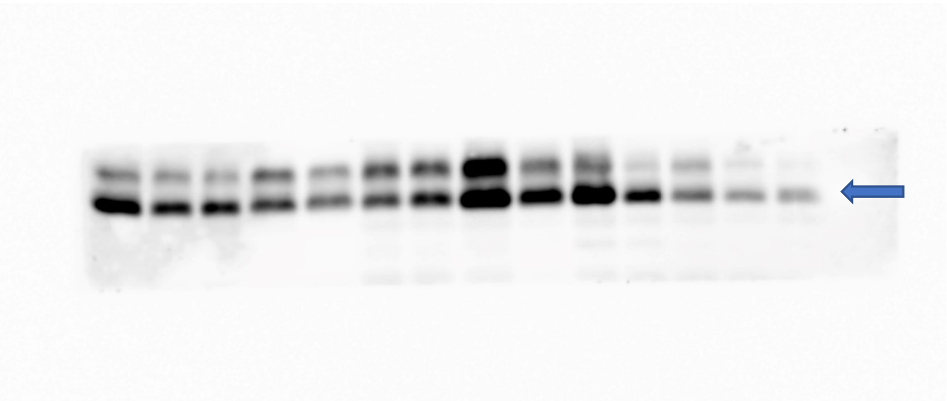

P38

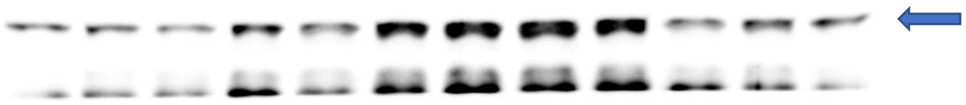

ERK1/2

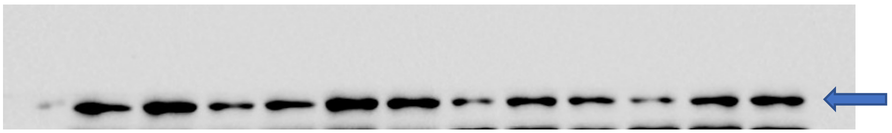

phospho- ERK1/2

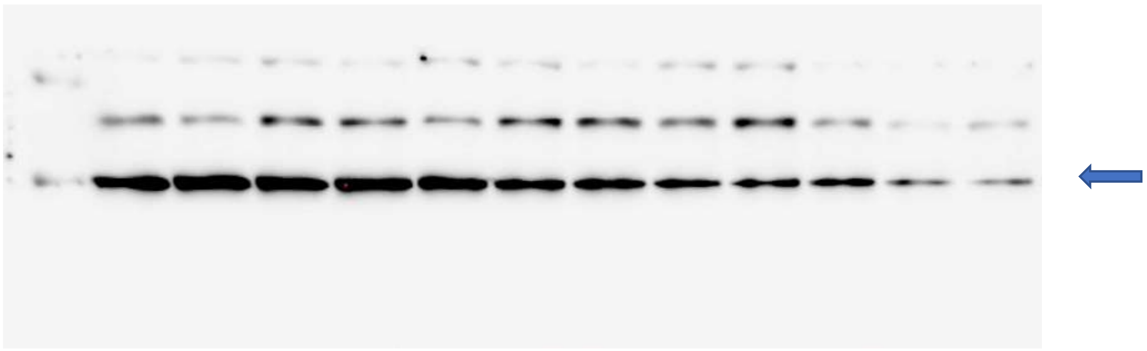

GSK-3α and GSK-3β

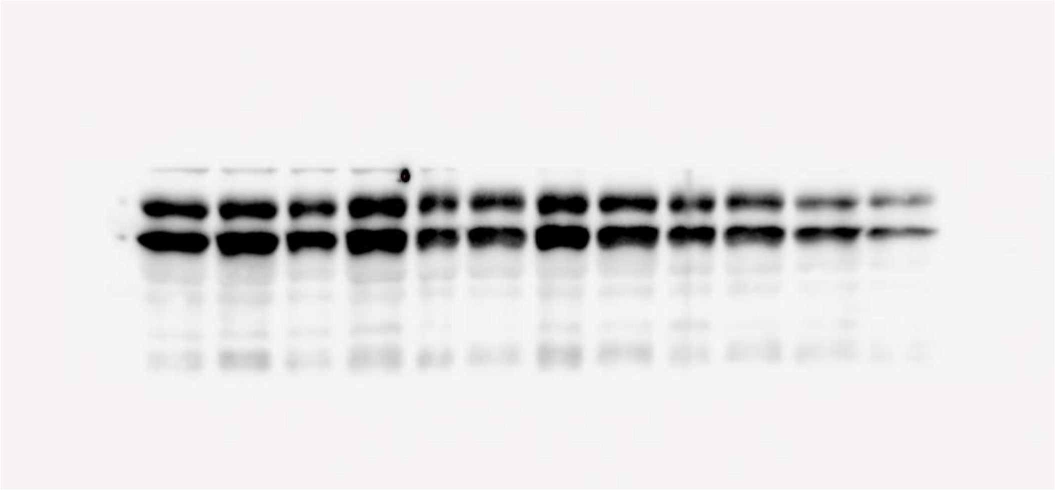

AKT-1/2/3

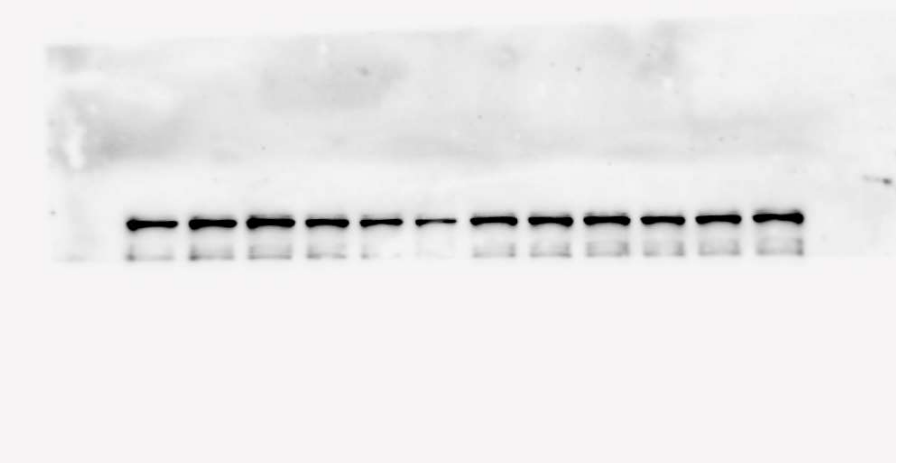

pAKT-1/2/3

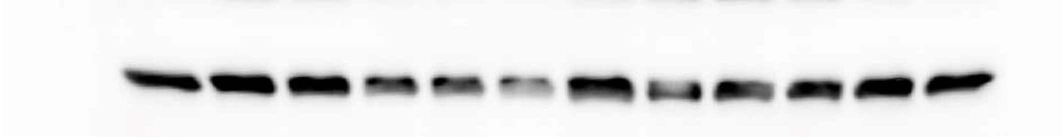

actin

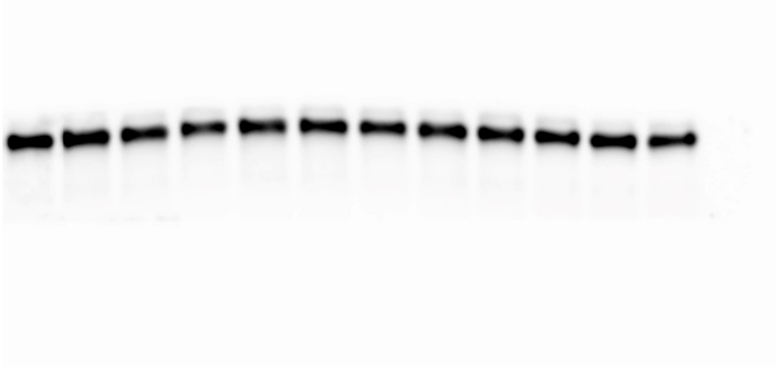

Figure 6A

C3aR1

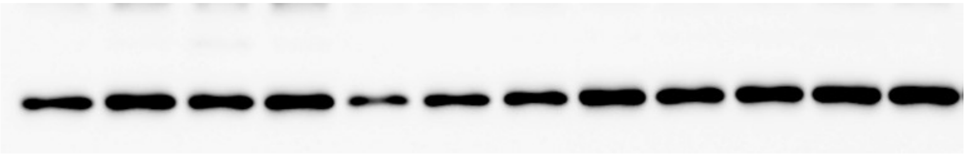

vinculin

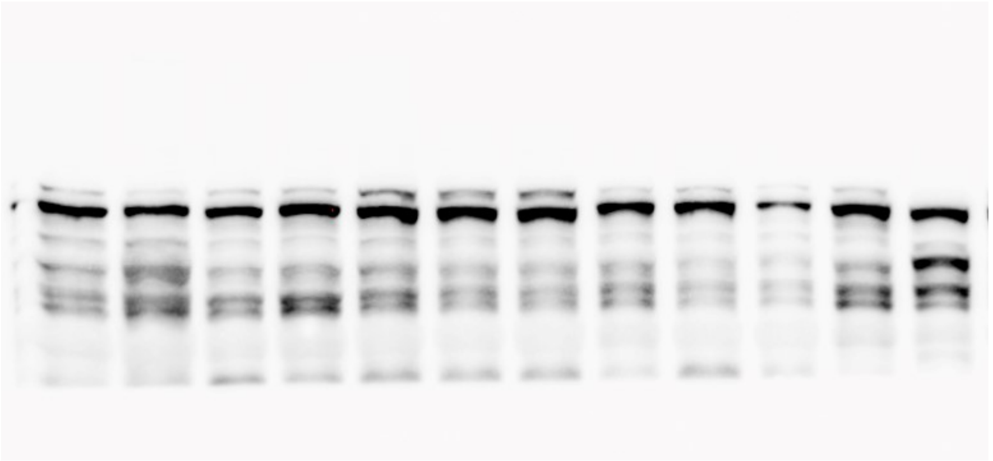

Figure 7E

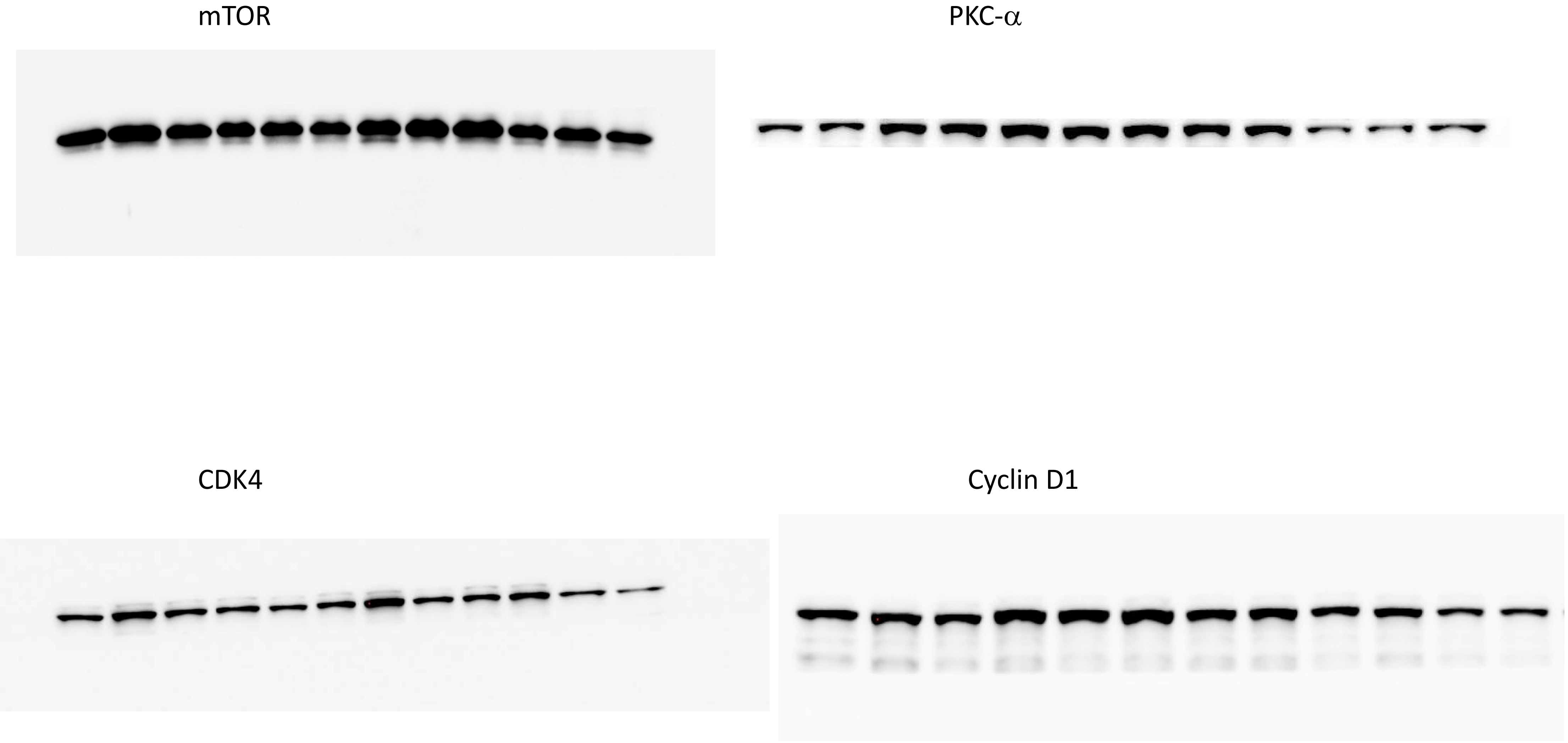

Cyclin E

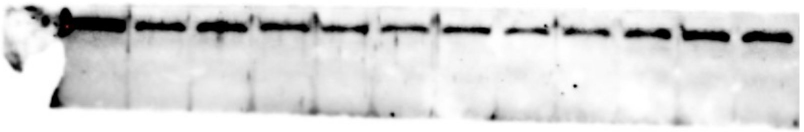

vinculin

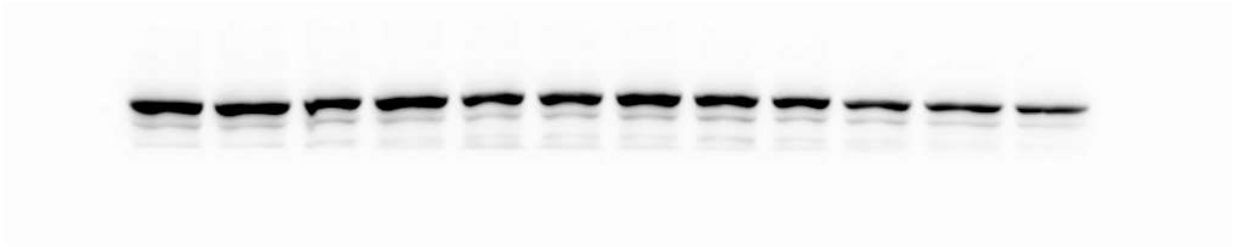

Figure 7F

OXPHOS

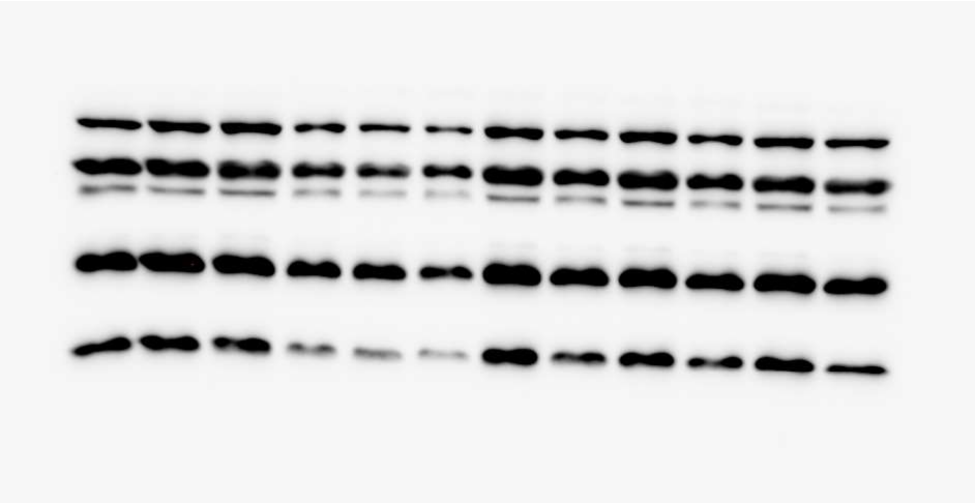

vinculin

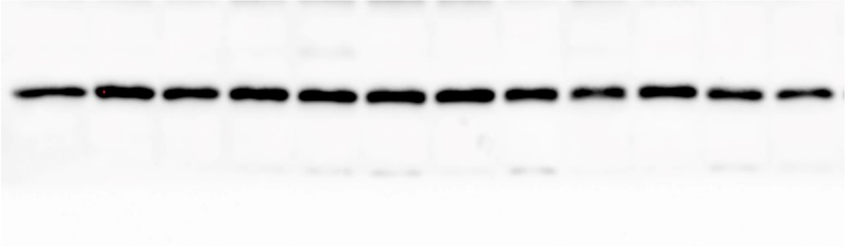

CV-ATP5A
CIII-UQCRC2
CIV-MTCO1
CII-SDHB
CI-NDUFB8

Figure 8D

MMP2

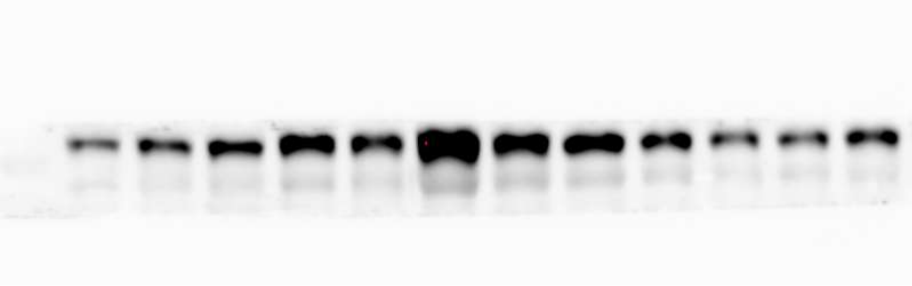

MMP9

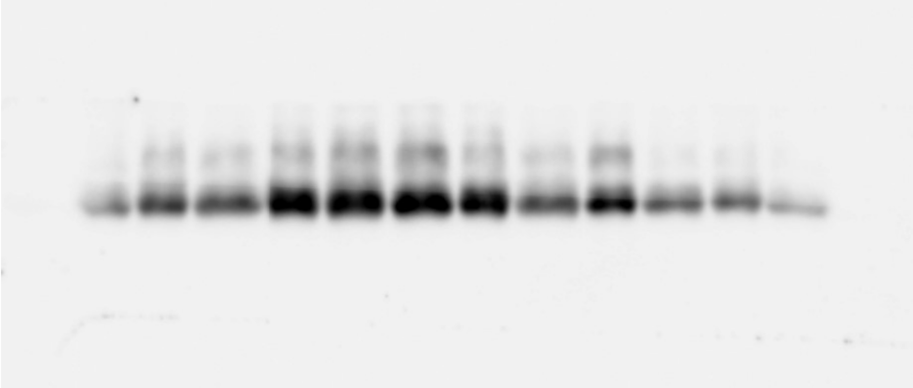

Adiponectin

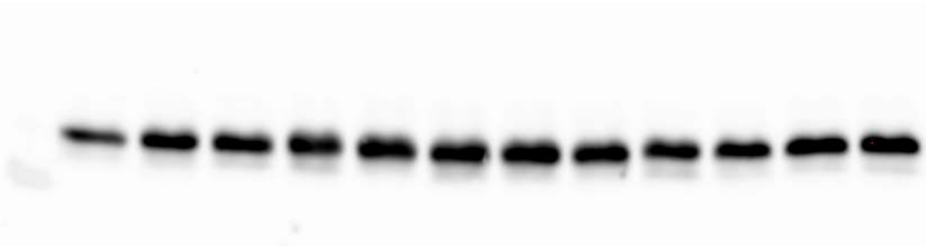

Perilipin

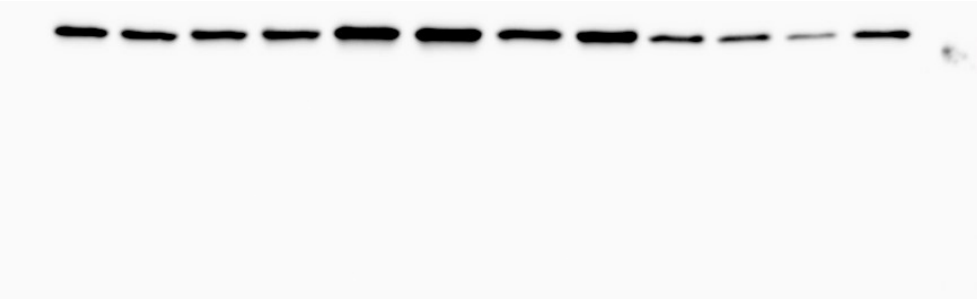

vinculin

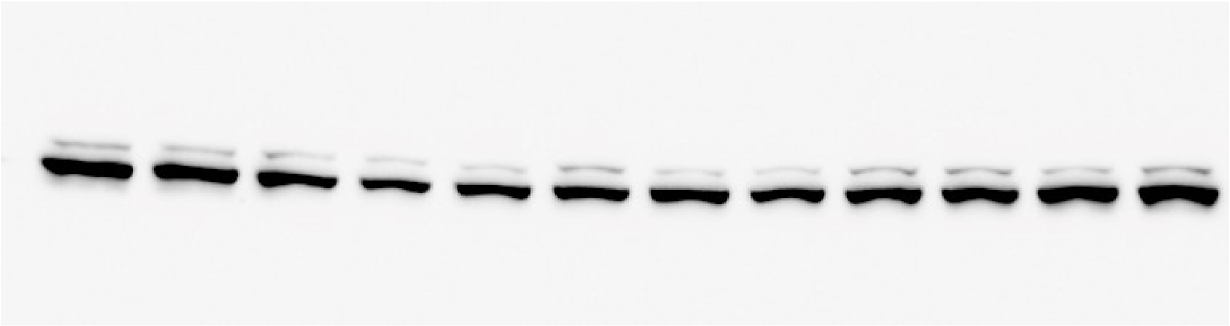

Figure 8E

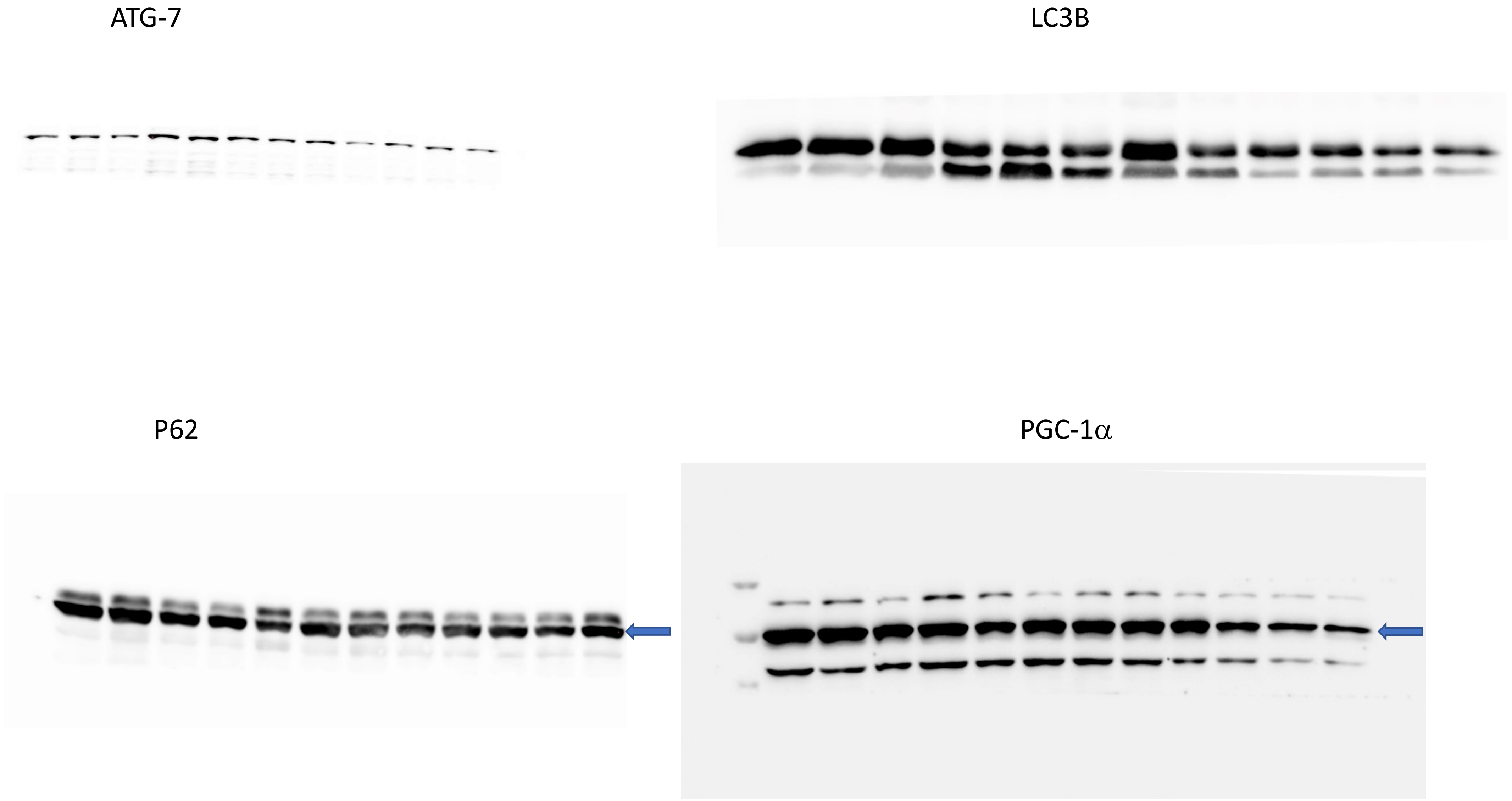

AMPK-α

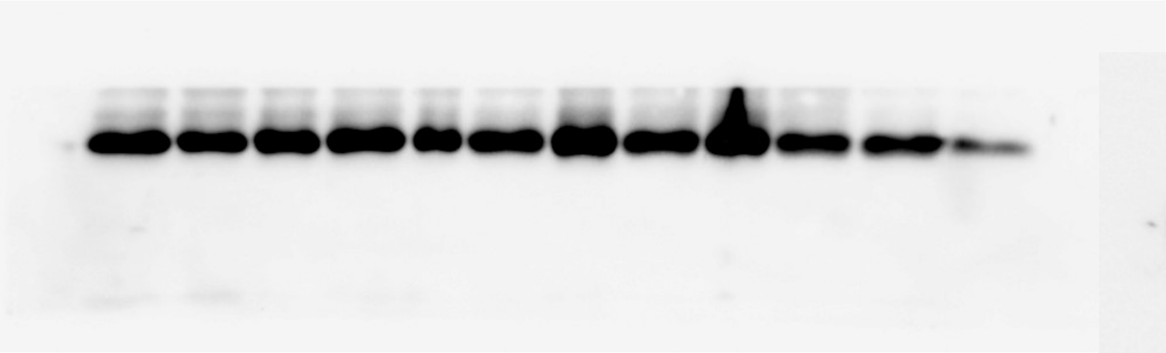

phospho AMPK-α

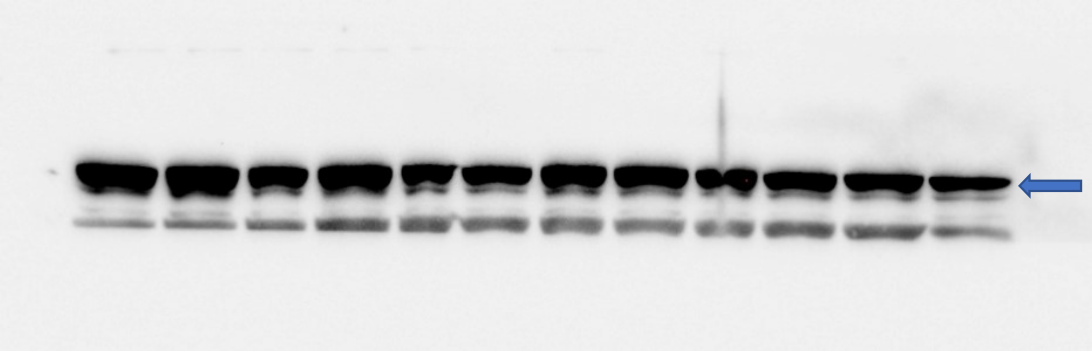

DRP-1

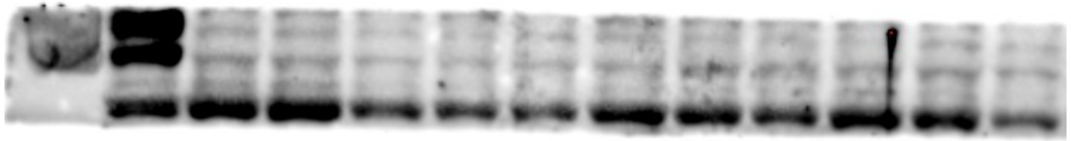

Vinculin

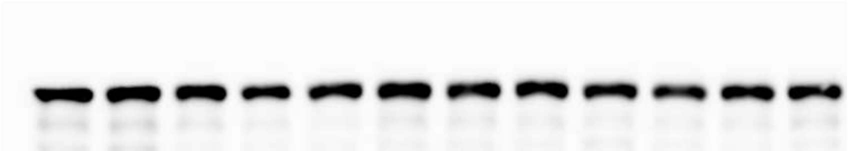

Supplementary Figure 4B

PSMB8

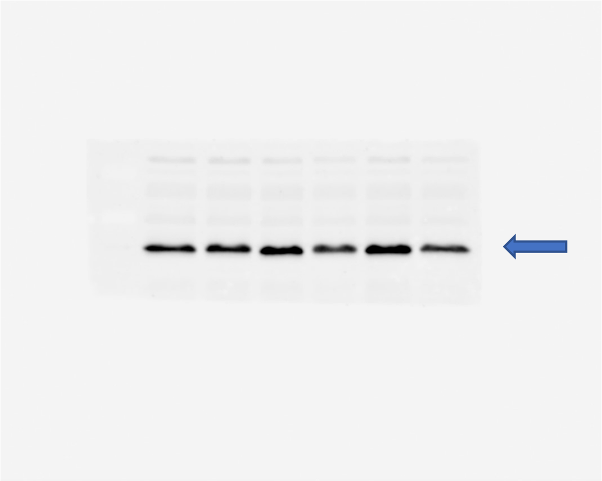

PSMB9

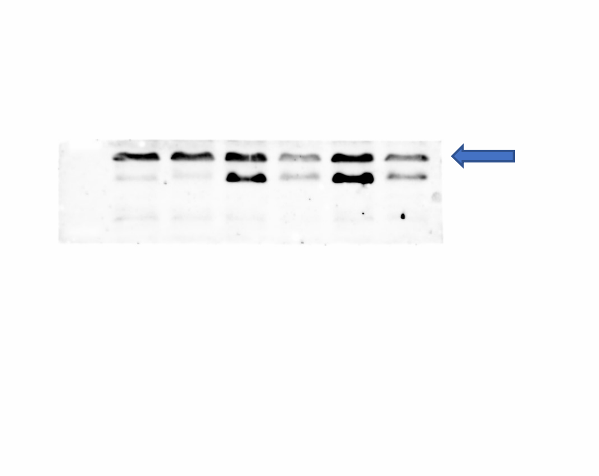

P62

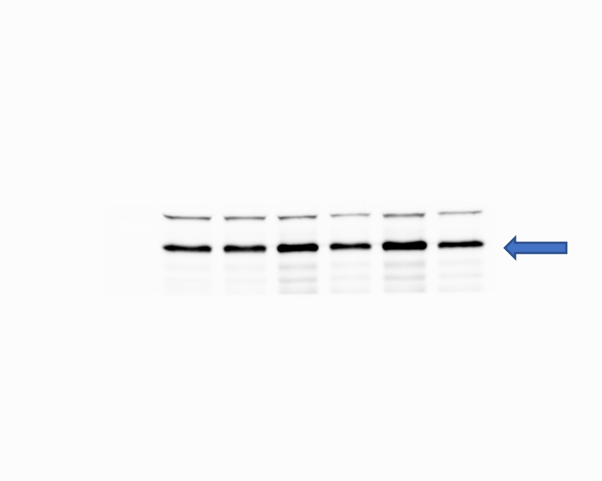

ATG-7

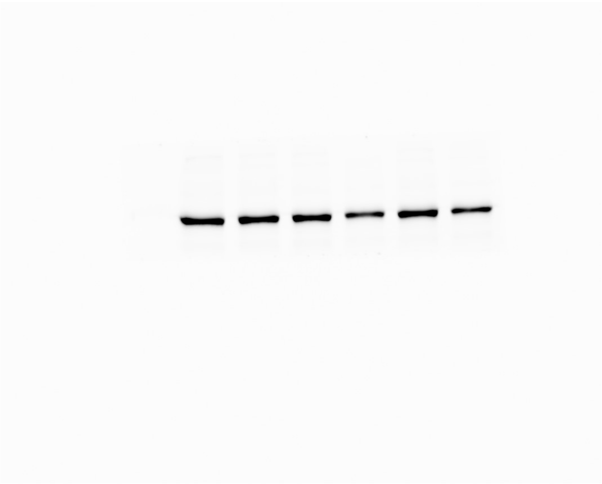

LC3-B

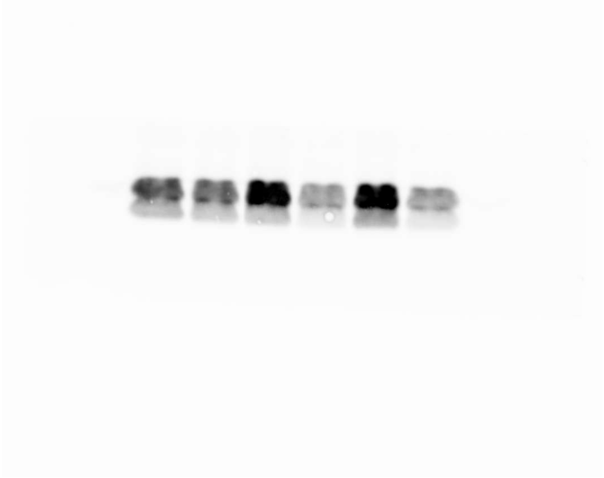

TLR-4

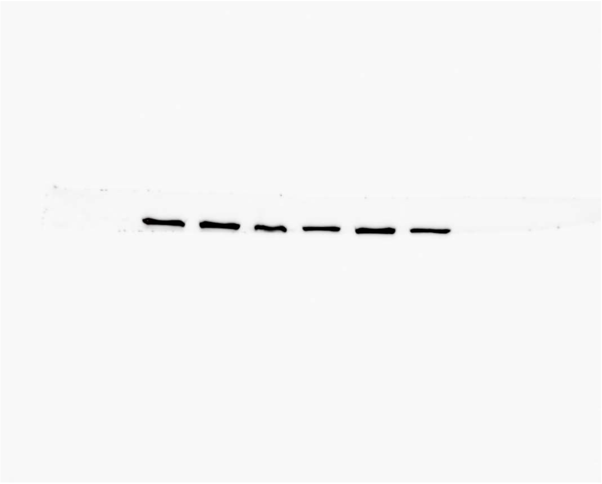

IKK-i

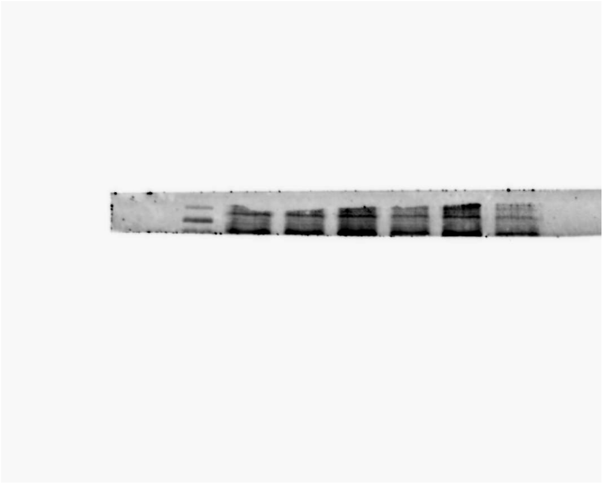

AKT1/2/3

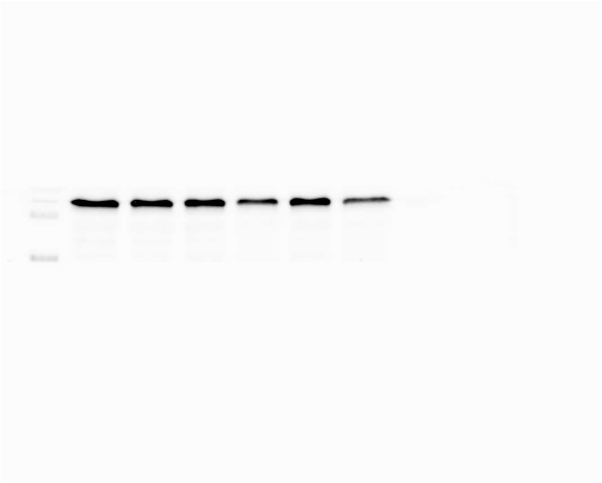

TNF-α

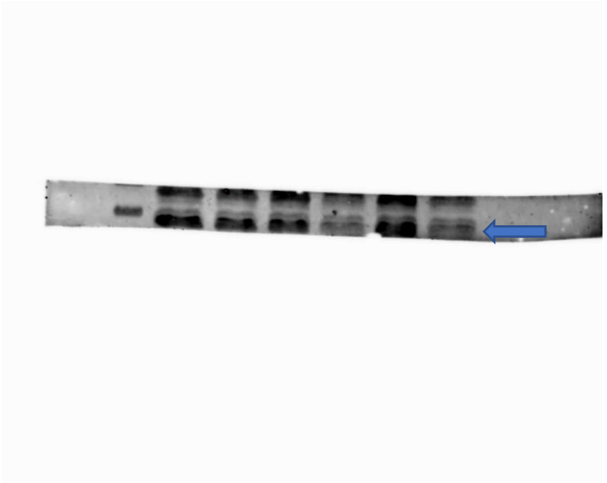

OXPHOS

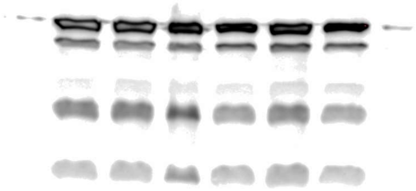

GAPDH

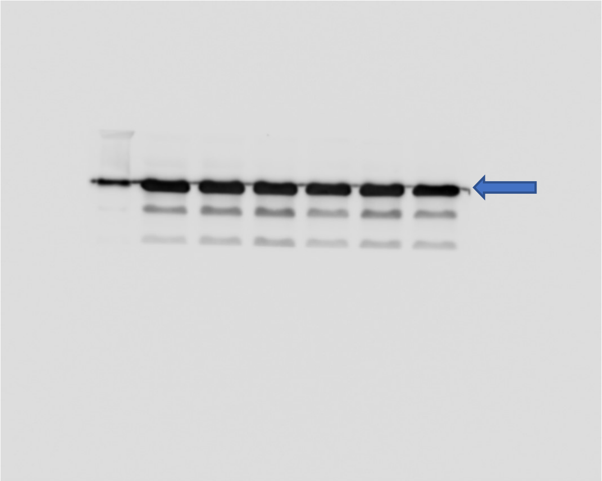

C3aR1

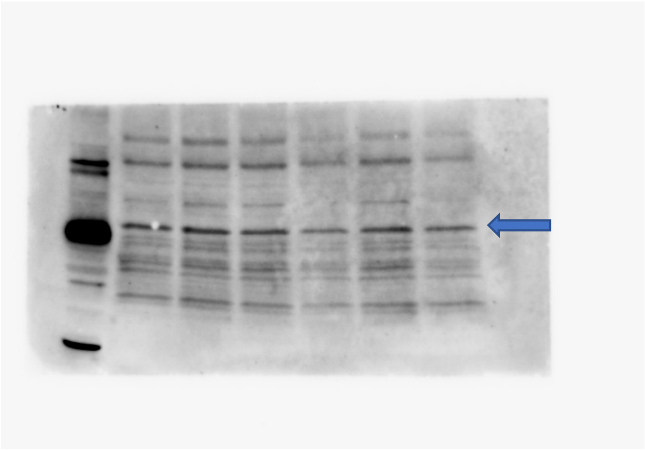

C5aR1

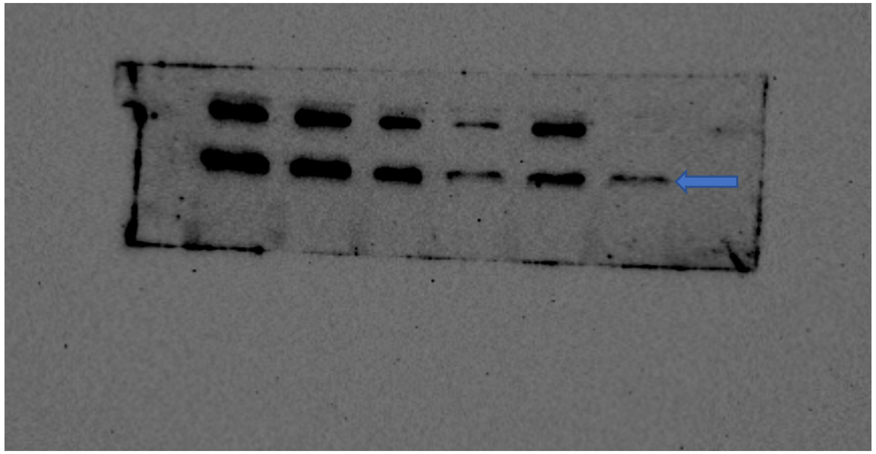

vinculin

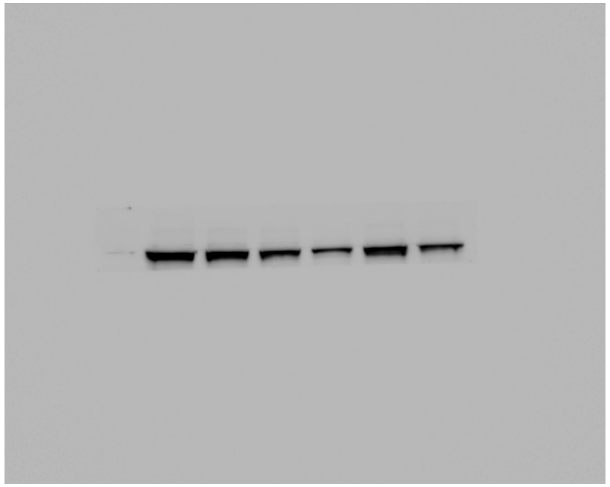

Supplement: Supplementary file 9 — Original Data File [file 41419_2022_5416_MOESM9_ESM.pdf]
